# From immunology to artificial intelligence: revolutionizing latent tuberculosis infection diagnosis with machine learning

**DOI:** 10.1186/s40779-023-00490-8

**Published:** 2023-11-28

**Authors:** Lin-Sheng Li, Ling Yang, Li Zhuang, Zhao-Yang Ye, Wei-Guo Zhao, Wen-Ping Gong

**Affiliations:** 1 Beijing Key Laboratory of New Techniques of Tuberculosis Diagnosis and Treatment, Senior Department of Tuberculosis, the Eighth Medical Center of PLA General Hospital, Beijing, 100091 China; 2 Hebei North University, Zhangjiakou, 075000 Hebei China; 3 Senior Department of Respiratory and Critical Care Medicine, the Eighth Medical Center of PLA General Hospital, Beijing, 100091 China

**Keywords:** Tuberculosis (TB), Latent tuberculosis infection (LTBI), Machine learning (ML), Biomarkers, Differential diagnosis

## Abstract

Latent tuberculosis infection (LTBI) has become a major source of active tuberculosis (ATB). Although the tuberculin skin test and interferon-gamma release assay can be used to diagnose LTBI, these methods can only differentiate infected individuals from healthy ones but cannot discriminate between LTBI and ATB. Thus, the diagnosis of LTBI faces many challenges, such as the lack of effective biomarkers from *Mycobacterium tuberculosis* (MTB) for distinguishing LTBI, the low diagnostic efficacy of biomarkers derived from the human host, and the absence of a gold standard to differentiate between LTBI and ATB. Sputum culture, as the gold standard for diagnosing tuberculosis, is time-consuming and cannot distinguish between ATB and LTBI. In this article, we review the pathogenesis of MTB and the immune mechanisms of the host in LTBI, including the innate and adaptive immune responses, multiple immune evasion mechanisms of MTB, and epigenetic regulation. Based on this knowledge, we summarize the current status and challenges in diagnosing LTBI and present the application of machine learning (ML) in LTBI diagnosis, as well as the advantages and limitations of ML in this context. Finally, we discuss the future development directions of ML applied to LTBI diagnosis.

## Background

Tuberculosis (TB) is an infectious disease caused by *Mycobacterium tuberculosis* (MTB) primarily affecting the respiratory system. The latest global TB report released by the World Health Organization (WHO) states that about 25% of the worldwide population has been infected with MTB [1]. Despite over a century of relentless endeavors to eliminate TB, this persistent infection continues to pose a significant menace to public health. The WHO’s Global Tuberculosis Report 2022 documented a staggering 10.6 million newly diagnosed cases and 1.6 million fatalities worldwide, solidifying TB as the foremost cause of death attributed to a solitary infectious agent [2].

**Fig. 1:**
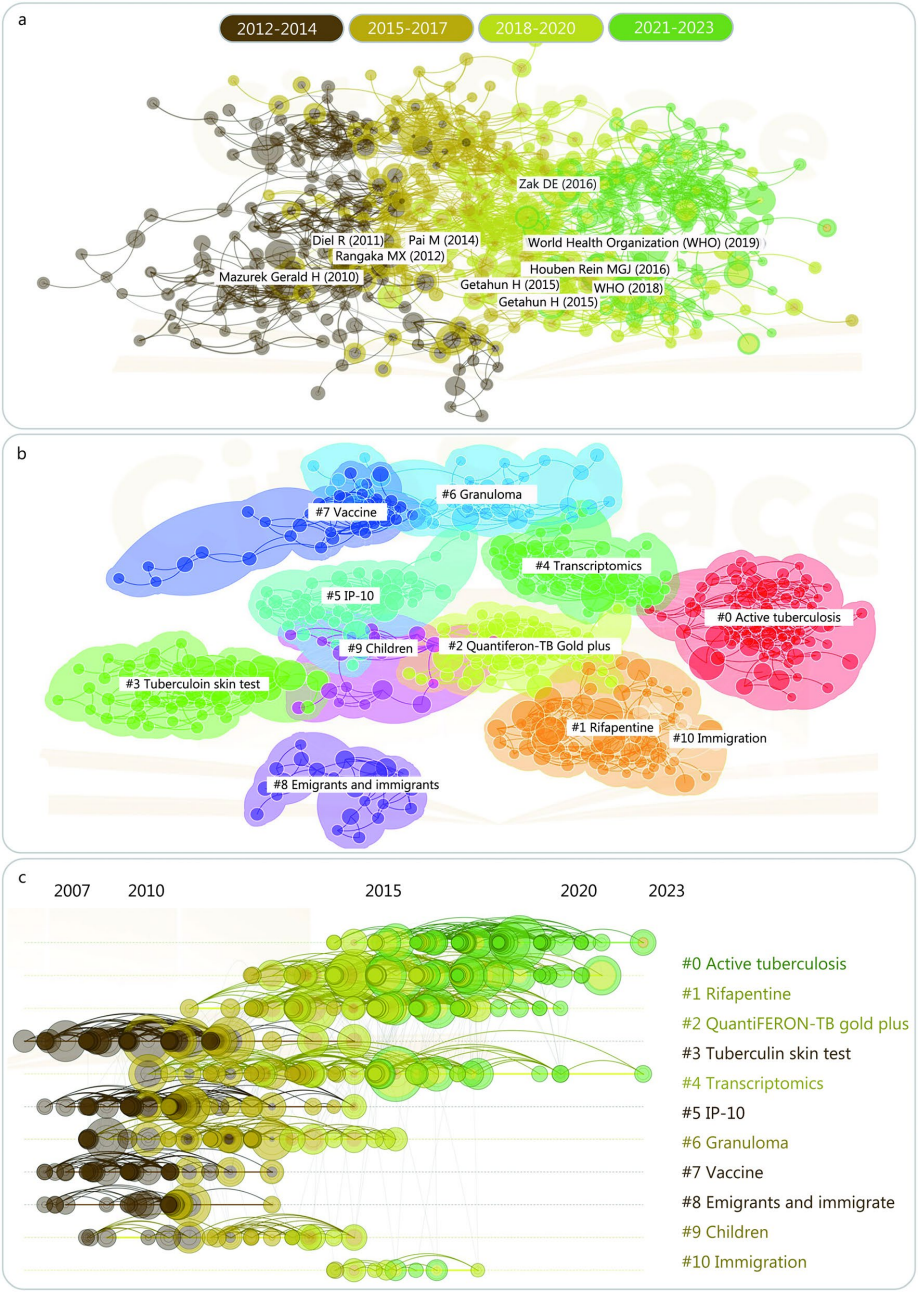
Bibliometric analysis of studies involved in latent tuberculosis infection (LTBI). In the Web of Science database, the search formula “{[TS = (tuberculosis)] OR [TS = (TB)]} AND {[TS = (latent tuberculosis infection)] OR [TS = (LTBI)]}” was used to retrieve and export the full record results (*n* = 2724). In addition, CiteSpace 6.2.R2 (64-bit) Basic (https://citespace.podia.com) was used to perform citation-based visualization of the data derived from Web of Science, including: **a** the research progress map with three years as time slices, **b** literature clustering based on keywords, and **c** the distribution of clusters on the timeline. IP-10 interferon protein-10, TS topic, TB tuberculosis

The difficulty in eliminating TB can be attributed to the diverse mechanisms of immune evasion and immune response manipulation by MTB [3]. MTB can persist in the human body for years without causing clinical symptoms, leading to a condition known as latent tuberculosis infection (LTBI) [4]. The global prevalence of LTBI was 24.8% (95% CI 19.7–30.0%) and 21.2% (95% CI 17.9–24.4%) based on interferon-gamma release assay (IGRA) and 10 mm tuberculin skin test (TST) cutoffs, respectively [5]. We conducted a comprehensive literature search in the PubMed database and depicted the most up-to-date research trends as depicted in Fig. 1. It illustrates the progressive shift in LTBI research focus, transitioning from initial macroscopic and pathological investigations to an immunological standpoint, which serves as the fundamental basis for the latest LTBI definition proposed by the WHO. LTBI is considered the primary source of new TB cases [6] and continues to be a major obstacle to achieving the WHO’s goal of ending the TB epidemic. Less than 25% of the global population infected with MTB will develop LTBI [7], and approximately 10% of those individuals will transition to active tuberculosis (ATB) at some point in their lives [8]. The lack of effective diagnostic tools to distinguish LTBI from ATB is a potential contributor to the high TB incidence and mortality rates [9, 10].

To address the diagnostic challenge of LTBI, recent advancements in machine learning (ML) technology have provided new avenues for distinguishing LTBI from ATB [11, 12]. ML algorithms and models offer a possibility for differential diagnosis of LTBI and ATB based on immunologic, imaging, and other biomarkers. In this review, we summarize the immune mechanisms of LTBI and commonly used diagnostic methods and focus on the latest developments of ML methods for LTBI diagnosis, including primary techniques, application scenarios, strengths, limitations, and future trends. We also discuss the major challenges facing this field, such as sample size, feature selection, and overfitting. ML has great potential to improve the accuracy and reliability of LTBI diagnosis, although the use of more rational data processing and analysis methods will be necessary. Such efforts will ultimately enhance the precision and accuracy of LTBI diagnosis, providing valuable support for TB control and treatment.

## MTB’s characteristics during latent infection

The understanding of LTBI has gone through 3 phases: the period of gross anatomy, the period of pathology, and the period of immunology [13]. Nowadays, LTBI refers to a condition where an individual has been infected with MTB but does not exhibit any symptoms or signs of active disease. Most people with LTBI have never had TB, however, about 5–15% of patients will progress to TB [14, 15]. The following section will discuss the characteristics of MTB during latent infection.

### Dormant state

During the period of LTBI, MTB enters a dormant or non-replicating state within the host [16]. A characteristic feature of this dormant state is the slowing down of bacterial metabolic activity. While the exact mechanisms and triggers of this dormant state remain incompletely understood, it is believed to be a survival strategy employed by the TB bacteria to evade the immune system. Within the host, MTB can exist as small clusters or individual bacilli within macrophages or other immune cells. This dormant state enables the mycobacteria to survive for long periods, even for years or decades, without causing active disease. During dormancy, MTB undergoes various physiological changes, leading to significant alterations in its antigen expression profile [17]. Previous studies have identified 124 antigens associated with LTBI, which have been categorized into 6 major classes based on their functions: dormancy survival regulon antigens (DosRs, *n* = 54), reactivation antigens (RAs, *n* = 20), nutrition starvation-associated antigens (*n* = 7), resuscitation-promoting factor antigens (Rpfs, *n* = 5), toxin-antitoxin system-associated antigens (*n* = 8), and other antigens associated with LTBI (*n* = 30) [18–21]. MTB downregulates genes associated with active replication and metabolism while upregulating genes associated with adaptation and stress response. These changes facilitate the survival of the TB bacteria in the immune microenvironment of the host.

### Asymptomatic nature

One of the main characteristics of LTBI is its asymptomatic nature. Individuals with LTBI do not exhibit any clinical symptoms related to patients with ATB [22]. The absence of symptoms in LTBI is attributed to the effective immune response that inhibits the replication and progression of the bacteria, preventing the development of the disease. The host’s containment of MTB primarily relies on the formation of granulomas. Granulomatous lesions are characteristic pathological changes in TB and exhibit heterogeneity in different stages of infection [23]. In the latent infection stage, granulomas show significant fibrosis around caseous necrotic nodules with minimal inflammation or calcification. These granulomas restrict the growth of MTB, maintaining the bacteria in a controlled state and establishing a host-bacteria equilibrium. Despite the absence of symptoms, individuals with latent pulmonary TB infection may test positive on diagnostic tests such as TST or IGRA [22]. These tests detect immune reactions to MTB proteins, indicating prior exposure to the bacteria.

### Reactivation risk

The reactivation of MTB in LTBI refers to the transition from a latent state to ATB. While the majority of individuals with long-term latent pulmonary TB never progress to ATB, a small proportion may experience reactivation at some point in their lives. The exact mechanisms of LTBI reactivation are not fully understood. Currently, it is widely believed that Rpfs play a crucial role in the activation process of MTB [24, 25]. MTB can express 5 Rpf proteins, namely RpfA (Rv0867c), RpfB (Rv1009), RpfC (Rv1884c), RpfD (Rv2389c), and RpfE (Rv2450c) [26]. The function of these proteins is to hydrolyze peptidoglycan, and their proposed mechanism involves enzymatically modifying the bacterial cell wall and promoting cell separation, thus contributing to the resuscitation of dormant MTB [27, 28].

The risk of reactivation depends on various factors. (1) Weakened immune system. The primary risk factor for reactivation is immune system impairment. Conditions such as human immunodeficiency virus (HIV) infection, immunosuppressive therapies (e.g., corticosteroids, chemotherapy), diabetes, certain cancers, and end-stage renal disease compromise the immune response, making it ineffective in controlling MTB. (2) Time since infection. The highest risk of reactivation occurs within the first two years after initial infection with MTB. However, the risk persists throughout life, albeit at a lower rate. Most reactivation cases occur within the initial few years, but some individuals may experience reactivation several years or decades after the primary infection. (3) Age. Infants, young children, and the elderly who have LTBI are at higher risk of reactivation, with an increased likelihood of developing ATB. This is attributed to their weaker immune system’s ability to control the infection. (4) Prior history of TB. Individuals who previously had ATB and completed treatment have a higher risk of reactivation compared to those without a history of TB.

## Immunological mechanisms of LTBI

### Innate immune responses induced by MTB

**Fig. 2:**
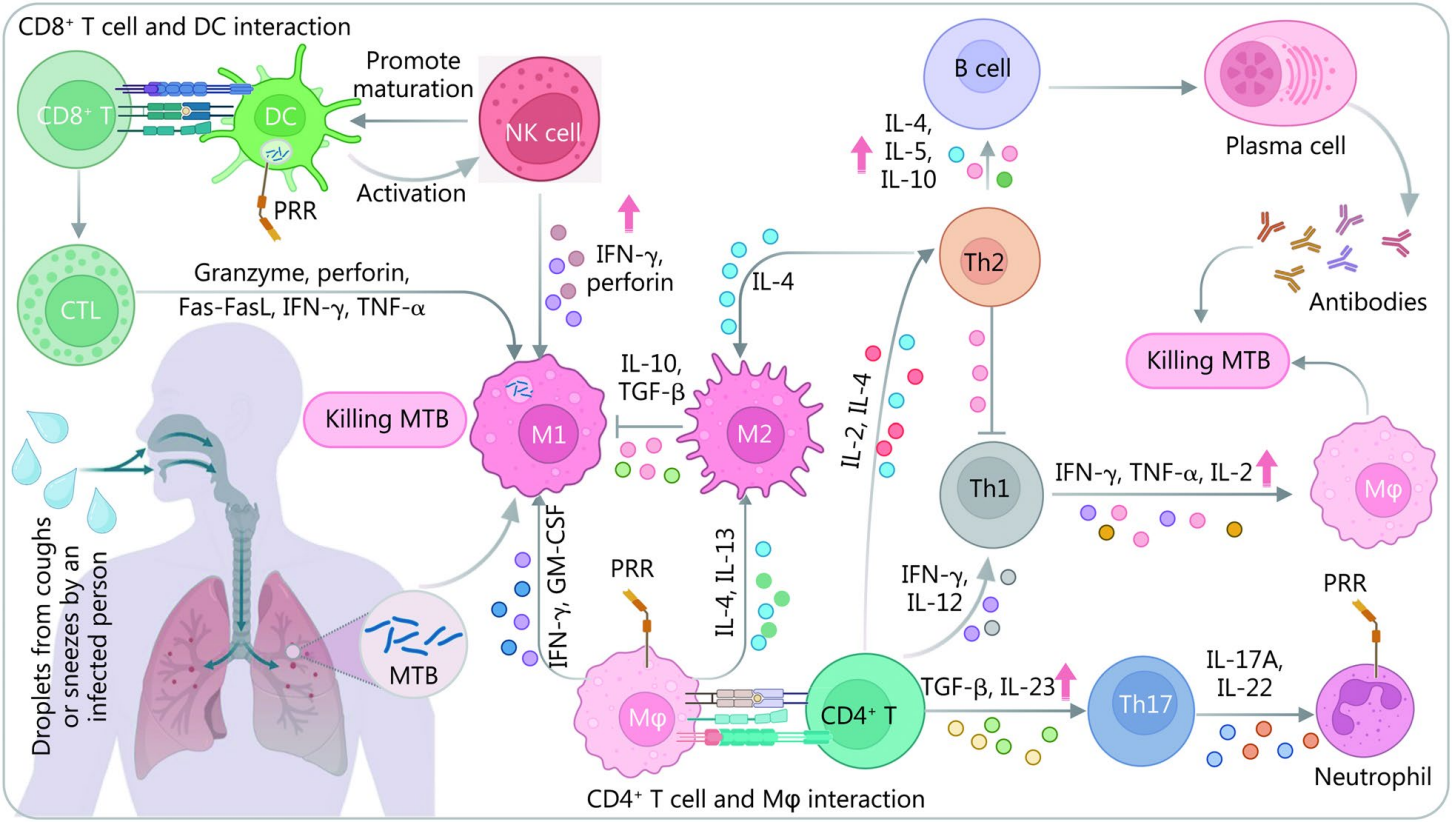
Overview of the mechanisms of innate and adaptive immunity in response to invasion by MTB in humans. Following MTB recognition by APCs, such as macrophages, antigens are presented to CD4^+^ T lymphocytes via MHC II molecules, and the activated CD4^+^ T lymphocytes will differentiate into Th1 (microenvironment with IFN-γ and IL-12), Th2 (IL-2 and IL-4), and Th17 (TGF-β and IL-23) cells. Th1 cells secrete IFN-γ and facilitate the clearance of MTB, while Th2 inhibits the action of Th1 and can also stimulate the production of antibodies by B cells to kill MTB. Th17 secretes cytokines that recruit neutrophils, macrophages, etc., to play an anti-inflammatory effect. NK cells promote the maturation of APCs, such as DCs, and can activate other immune cells, including macrophages and CTLs. Polarization of macrophages in different cytokine environments results in different immune effects. The complexities of host immunity to MTB highlight the need for further research to better understand the underlying mechanisms of host defense response. APCs antigen-presenting cells, CTL cytotoxic T lymphocyte, MHC II histocompatibility complex II, IFN-γ interferon-γ, IL interleukin, Mφ macrophage, PRR pattern recognition receptor, Th cells helper T cells, TGF transforming growth factor, DC dendritic cell, NK cell natural kill cell, Fas-FasL Fas and Fas ligand, TNF tumor necrosis factor, MTB *Mycobacterium tuberculosis*, GM-CSF granulocyte–macrophage colony-stimulating factor, M1 type I macrophage, M2 type II macrophage

LTBI is a state in which the host is infected with MTB but does not progress to ATB. The body immobilizes MTB at the site of infection to initiate an anti-infective process. During this process, the innate immune system serves as the first line of defense against MTB infection [29]. The innate immune system is a defense system that has evolved gradually during the long-term evolution of germline cells and is primarily composed of tissue barriers, innate immune cells such as natural killer (NK) cells, inflammatory macrophages, eosinophilic granulocytes, and innate immune molecules such as complement proteins, and recombinant molecules (Fig. 2). These cells play a crucial role in clearing bacteria such as MTB [30]. Therefore, understanding the defense process involving diverse innate immune cells is essential for comprehending the immunological mechanisms of LTBI and identifying potential biomarkers for differential diagnosis. Here, we focus on the primary innate immune cells related to LTBI and their roles, including macrophages, neutrophils, dendritic cells (DCs), and NK cells.

#### Macrophages

Alveolar macrophages (AMs) are the earliest cells to initiate an immune response against pathogens entering the respiratory tract and play a crucial role in the early and chronic phases of MTB infection [31, 32]. Macrophages have a variety of ways to kill and eliminate MTB, including phagocytosis and lysosomal degradation of acid enzymes, autophagy, apoptosis, reactive oxygen species (ROS), and nitrogen release [33].

When MTB enters the lungs, it first encounters alveolar epithelial cells, AMs, and the mucus clearance barrier, which activates macrophages through different pathogen-associated molecular patterns (PAMPs) and pattern recognition receptors (PRRs) pathways to produce different killing effects [34]. For a long time, MTB infection was characterized pathologically by granulomatous inflammation, featuring the aggregation of various immune cells, including foam macrophages (a type of macrophage with lipid droplets) that suppress inflammation, diminish antigen presentation capabilities, and phagocytic functions [35]. Furthermore, macrophages within granulomas can express 2 critical enzymes, nitric oxide and arginase-2 (NOS2), and arginase-1 (Arg1), both of which can generate ammonia metabolites to regulate the response against MTB [36]. It has been reported that M2 macrophages inhibit inflammatory responses, and this inhibition mechanism is attributed to Arg1, which competes with NOS2 in granulomas to suppress macrophages’ killing effects against MTB [37, 38]. Another cell that inhibits the response against MTB is mesenchymal stem cells, which can promote the transformation of M1 macrophages into M2 macrophages, thereby suppressing inflammatory responses [39]. The inhibitory effect of M2 macrophages on inflammation in granulomas helps alleviate lung damage. Additionally, previous study suggests that an interaction between MTB and AMs may induce apoptosis in AMs via the action of tumor necrosis factor-α (TNF-α), thereby increasing the survival rate of MTB [40].

Therefore, we need to conduct large-scale research to determine whether macrophage modulation can be achieved to control MTB early. Overall, macrophages play a crucial role in MTB infection, particularly AMs that initiate an immune response early on and combat pathogens through multiple clearance mechanisms. Furthermore, in the chronic phase of MTB infection, macrophages still play an essential role, with foam macrophages, M1 macrophages, and M2 macrophages generating different effects that affect inflammation and MTB clearance differently. Thus, in the differential diagnosis of LTBI, surface markers and metabolic products of macrophages may become a new strategy worthy of our attention and further research.

#### DCs

In the context of DCs, an essential player in the immune system, it is worth noting that granulocyte–macrophage colony-stimulating factor (GM-CSF) appears early in T cell secretion upon infection with MTB, and it plays a significant role in promoting the differentiation of monocytes (moDCs). These differentiated moDCs are instrumental in driving the adaptive immune response during the initial phase of infection, resulting in the production of protective T cell responses within granulomas [41]. Consequently, the function of DCs becomes crucially important in providing critical protection for controlling MTB infection.

#### Neutrophils

Neutrophils are the first responders of the innate immune system against invading pathogens [42]. However, when infected with MTB, neutrophils are found to not directly phagocytose the bacterium. Instead, they are attracted to the granulomas formed by infected macrophages [43]. The reason for this is that neutrophils utilize the phagocytosis of granulomas and the nicotinamide adenine dinucleotide phosphate-mediated ROS system to kill MTB [44]. Despite playing a crucial role in killing MTB, an excessive amount of neutrophils may lead to lung tissue damage [45]. This is because, during a respiratory burst, neutrophils release a large amount of cell factors such as peroxidase, elastase, and collagenase [46]. These results suggest that while neutrophils play an important role in immune response, an excessive inflammatory response can have negative effects. In fact, neutrophils may serve as transporters of MTB, transporting it to other tissues in the body [47], thus inducing TB infection. Additionally, a study has shown that MTB can induce cell death by inhibiting prostaglandin E production [48]. Currently, the region of deletion 1 (*RD1*) gene has been confirmed to be important for the virulence of MTB in the body [49].

#### NK cells

NK cells are essential components of the innate immune response and play a crucial defensive role in the early stages of MTB invasion [50]. NK cells are primarily derived from bone marrow and can secrete various cytokines, among which interferon-γ (IFN-γ) is one of the most important. Previous studies have reported that IL-12 and IL-18 are also important factors for stimulating NK cells to release IFN-γ [51, 52]. These cytokines can enhance immune responses and effectively support NK cells in combating MTB infection. Research has suggested that DCs can activate NK cells at the site of inflammation located in the secondary lymphoid organs, thereby controlling infections and activating CD4^+^ T cells to release IFN-γ in response to inflammatory reactions, particularly MTB infection [53]. Moreover, there are differences in the phenotypes of NK cells among ATB patients, individuals with LTBI, and healthy controls. Recently, Albayrak et al. [50] classified NK cells found in the blood into three types based on their phenotypes, including CD56^bright^, CD56^dim^, and CD56^neg^ NK cells. They found that the ratio of total NK cells and CD56^neg^ NK cells was lower in individuals with ATB than in those with LTBI. Furthermore, the ratio of CD56^dim^ NK cells was higher in individuals with LTBI, and the amount of IFN-γ produced in vitro was higher in individuals with LTBI than in those with ATB [50]. Similarly, other study has found that the number of CD56^dim^CD16^+^ and CD56^dim^CD27^+^ NK cells was significantly higher in individuals with LTBI than in ATB patients [54]. These findings suggest that different states of TB infection are associated with the diversity of NK cells, and these newly discovered associations will further highlight the potential biomarkers for differential diagnosis of LTBI and ATB.

### Adaptive immune responses induced by MTB

Adaptive immunity, facilitated by the activity of T and B lymphocytes, is responsible for the immune response against infections. Upon exposure to the antigen of MTB, T and B cells generate an immune response that primarily encompasses humoral immunity facilitated by B cells and cellular immunity facilitated by T cells in order to provide protection against MTB (Fig. 2).

#### *CD4*^+^*T lymphocytes*

T cells are central immune cells vital in TB immunity in the human body. CD4^+^, CD8^+^, and Th17 cells are well-defined subsets of T cells. Although different T cell subtypes play varying roles in defending against MTB infection, CD4^+^ T cells are the primary cells in combating MTB. Upon MTB’s entry into the body, it is engulfed by macrophages and processed into antigen peptide complexes. These complexes are then presented to MHC class II molecules, which help stimulate the activation and differentiation of CD4^+^ T cells, particularly Th1 cells. Th1 cells play vital roles in inducing cytokines from macrophages, primarily IL-2, IFN-γ, and TNF-α, which help combat MTB within infected cells. Simultaneously, Th1 cells produce ROS and reduce the effect of MTB within phagosomes [55]. Th2 cells, on the other hand, secrete IL-10, which reduces the expression of co-stimulatory molecules CD40 and CD86 on monocytes and macrophages, thus, affecting the antigen presentation process [56, 57]. Similar to Th1 cells, Th17 cells produce crucial cytokines such as IL-17, which are vital in fighting different pathogens and have also been proven to be important in TB defense [58, 59]. Furthermore, IL-17 can activate T cells to fight against MTB [60].

#### *CD8*^+^*T lymphocytes*

In contrast to CD4^+^ T cells, CD8^+^ T cells have traditionally been considered to have a minor impact on the prevention of MTB infection. However, recent studies have challenged this notion and suggested that the role of CD8^+^ T cells in fighting MTB infection may be more important than anticipated [61–63]. CD8^+^ T cells’ receptors accept the MHC I molecule complex, allowing them to differentiate into cytotoxic T lymphocytes (CTLs), which in turn secrete granzymes, perforin, IFN-γ, TNF-α, and other substances to promote macrophages to kill MTB [61, 64]. These findings suggest that the role of CD8^+^ T cells in TB immunity warrants further in-depth investigation. Nonetheless, we must remain cautious in generalizing laboratory results gained through animal experimentation to models of human infection. The cytokines, granules, and other substances utilized by CD8^+^ T cells in combating MTB require further examination. Additional independent research is required to more firmly establish the extent of the role of CD8^+^ T cells in TB immunity.

#### *The association between CD4*^+^*and CD8*^+^*T lymphocytes*

The above content demonstrates the crucial role of T cells in TB immunity. Different types of T cells produce distinct immune responses to MTB, highlighting the need to explore the role of CD8^+^ T cells, in addition to the well-established role of CD4^+^ T cells in TB immunity. Thus, understanding the overall nature of immune responses requires coordinating different types of T cells with other immune cells. For example, Th1 cells secrete cytokines such as IFN-γ and TNF-α, which induce macrophages to kill MTB. Similarly, CD8^+^ T cells can differentiate into CTLs to enhance the bactericidal effect of macrophages by producing cytokines. Furthermore, Th17 cells play a critical role in inflammatory responses by producing IL-17 and recruiting neutrophils, macrophages, and other immune cells to the site of inflammation.

#### The disparities in T cell immune responses between ATB and LTBI

There are differences in T cell immune responses between ATB and LTBI [13]. In LTBI, the characteristic of the T cell immune response is the presence of specific T cells that can recognize MTB antigens without causing evident disease. These T cells are often in an immune-controlled state, maintaining the infection in a latent state. In contrast, ATB is associated with immune dysregulation, characterized by excessive inflammation, tissue damage, and the appearance of symptoms. A recent study assessed the differences in the production of 40 cytokines/inflammatory factors in peripheral blood mononuclear cells from individuals with ATB and LTBI upon stimulation with different MTB peptides [65]. The study finding revealed significantly higher levels of interleukin-1 receptor antagonist (IL-1RA) in the cell culture supernatant of ATB patients compared to LTBI individuals. IL-1RA, a member of the IL-1 family, shares receptors with IL-1 but functions by inhibiting the biological activity of IL-1 [66]. The elevation of IL-1RA level suggests that the body generates more IL-1RA in ATB patients to counterbalance excessive IL-1 activity, potentially contributing to mitigating the inflammatory response. We have previously observed that the levels of TNF-α, induced by the novel vaccine candidate PP19128R, were higher in patients with ATB compared to individuals with LTBI [67]. Furthermore, we found that the number of IFN-γ^+^ T lymphocytes induced by MTB peptides was significantly elevated in mice with ATB when compared to those with LTBI infection [68]. These research findings indicate the existence of differential cytokine/inflammatory factor expression between ATB and LTBI, which may reflect distinct immunological characteristics in their immune responses. Further investigations are warranted to enhance our understanding of the development and treatment of TB.

#### T cell exhaustion or dysfunction during MTB infection

Despite the crucial role of T cells in killing MTB, chronic MTB infection can induce T cell exhaustion and dysfunction due to sustained antigen stimulation. Animal experiments have shown that continuous MTB antigen stimulation leads to a decrease in antigen-specific production of IFN-γ and IL-2 in mice and a reduction of memory CD8^+^ T cells, and overexpression of programmed death receptor 1 (PD-1), resulting in ineffective control of MTB infection [69]. Similarly, active pulmonary TB patients also exhibit T cell exhaustion and dysfunction under sustained MTB antigen stimulation, characterized by reduced production of MTB-specific INF-γ, TNF-α, and IL-2 by CD4^+^ and CD8^+^ T cells, as well as increased expression of PD-1 and its ligands on T cells, monocytes, macrophages, and B cells [70]. Recently, Pan et al. [71] identified 12 genes through single-cell sequencing that may be associated with exhaustion of CD4^+^ and CD8^+^ T cells following *Mycobacterium* infection, including *RPS26*, *ITM2C*, *GZMK*, *IL32*, *HLA-DRB1*, *TNFAIP3*, *JUN*, *ZFP36L2*, *GTF3C1*, *ZFP36*, *MT2A*, and *HOPX*. Among the features of T cell exhaustion and dysfunction caused by chronic MTB infection, immune checkpoints have gained increasing attention. In the case of MTB infection, blockade of immune checkpoint molecules may help enhance T cell responses and improve infection control [72]. Currently, the immune checkpoint proteins studied in the context of MTB infection include PD-1 [73], T cell immunoglobulin domain and mucin domain-3 (TIM-3) [74], cytotoxic T lymphocyte-associated antigen-4 (CTLA-4) [75], lymphocyte activation gene-3 (LAG-3) [76], and glucocorticoid-induced TNF receptor (GITR) [77]. Among them, PD-1/PD-L1 inhibitors (such as sintilimab) have become a research hotspot in recent years for immunotherapy of TB [78, 79], while TIM-3, CTLA-4, LAG-3, and GITR are promising targets for the next generation of immune therapies.

#### The role of B cells and humoral immunity

During the late 19th century, attempts were made to employ serum therapy for the treatment of TB. However, the lack of standardized protocols and reagents resulted in inconsistent research outcomes, leading to skepticism regarding the significance of humoral immunity in controlling MTB [79–81]. Recent studies indicate that B cell- and antibody-mediated immunity are instrumental in facilitating cellular immune responses, producing neutralizing toxins and antibodies, and forming memory [82, 83]. Some B cells capture antigens and stimulate CD4^+^ T lymphocytes to produce cell cytokines against MTB [84]. Others differentiate into B effector (Be-1 and Be-2) cell subgroups, which synthesize different pro-inflammatory cytokines based on the effector T lymphocytes they interact with [85]. Memory B cells play a pivotal role in thwarting reinfection by the same pathogen and are the foundation of the Bacillus Calmette–Guérin (BCG) vaccine’s efficacy [86].

Furthermore, recent research has revealed the intricate influence of IL-10 on B lymphocytes. IL-10 possesses immune-suppressive properties, capable of inhibiting the activity of various immune cells including B cells [87, 88]. This inhibition encompasses vital processes essential for generating effective immune responses, such as B cell proliferation, antibody production, and class switching [89–91]. However, under certain circumstances, IL-10 can induce the differentiation of B cells. Research observation has revealed that IL-10 can promote the differentiation of memory B cells into plasma cells [92], which are responsible for antibody secretion. Additionally, IL-10 plays a noteworthy role in regulating the balance among different subgroups of B cells. Specifically, it promotes the generation of regulatory B cells (also known as Bregs) possessing immunosuppressive functions. These Bregs exhibit the ability to modulate immune responses through various mechanisms, including inhibiting the activity of other immune cells and facilitating the formation of an anti-inflammatory environment [93, 94]. Within the realm of Bregs research, the most prominent cellular subgroup is B10 cells. Recent investigation has discovered that B10 cells suppress immune responses by expressing the immune-inhibitory cytokine IL-10 [95]. This finding further substantiates the significance of Bregs in immune regulation.

In addition to B cells, antigen-specific antibodies against MTB have also gained attention in the scientific community. As early as 2005, Roy et al. [96] demonstrated that treatment of MTB-infected mice with a single-cycle high-dose intravenous immunoglobulin (hdIVIg) greatly reduced bacterial burden in both the spleen and lungs, regardless of whether it was administered during the early or late stages of infection. Another study has demonstrated that the protective effect of immunoglobulin against TB in mice is dependent on the glycosylation of IgG [97]. Interestingly, in addition to mammals, evidence of a protective effect of MTB-specific antibodies has been found in humans. Li et al. [98] evaluated the protective effect of MTB-specific antibodies in 48 healthcare workers and 12 ATB patients. These results revealed that the antibodies from 7 of the healthcare workers exhibited a moderate protective effect against MTB, whereas the antibodies from the ATB patients showed no protective effect. Interestingly, further investigations indicated that 4 out of these 7 healthcare workers had evidence of LTBI [98]. A meta-analysis was conducted to investigate the extent to which LTBI reduces the risk of disease progression following re-exposure and re-infection (total *n* = 19,886) [99]. The result revealed that individuals with LTBI had a 79% lower risk of developing TB compared to individuals who were not infected. Similarly, other studies have also shown that antibodies from individuals with LTBI provide better protection in macrophage infection models compared to antibodies from ATB patients [100]. These data suggest that individuals with LTBI may produce certain protective MTB-specific antibodies that help combat MTB infection and prevent progression to ATB.

Currently, TB vaccine research primarily focuses on eliciting cell-mediated immunity. However, it is important to note that B cell-mediated humoral immune responses also contribute to the prevention of TB [101]. A study conducted by Lu et al. [100] utilized an unbiased antibody profiling approach and discovered that individuals with LTBI and ATB exhibit different MTB-specific humoral responses. Specifically, LTBI is linked to distinctive antibody Fc functional profiles, selective binding to Fc gamma receptor III (FcγRIII), and distinct antibody glycosylation patterns. Importantly, antibodies from LTBI were found to enhance phagolysosomal maturation, inflammasome activation, and macrophage killing of intracellular MTB when compared to antibodies from individuals with ATB [100]. The findings of this study have significant implications for vaccine development strategies, as the interaction between IgG Fc and Fcγ receptors is crucial for immune response regulation [102]. By understanding the Fc functional characteristics of MTB-specific antibodies and their selective binding to Fcγ receptors, researchers can more effectively target and design vaccines against TB. These findings suggest that B cell-mediated humoral immune responses play a relevant role in the control and elimination of TB infection.

### Signaling pathways

**Fig. 3:**
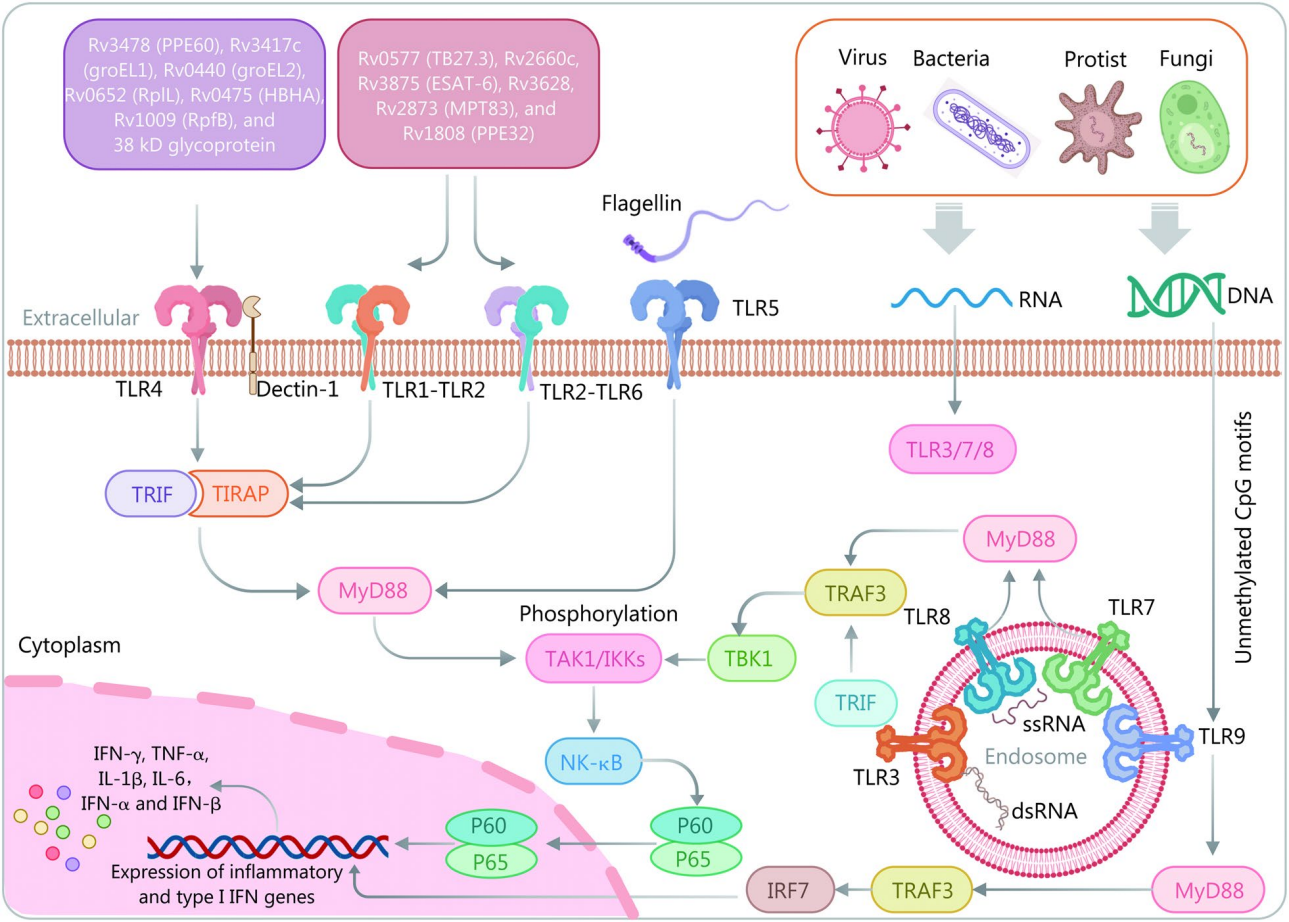
Immune signaling pathways involved in MTB infection in vivo. MTB infection triggers immune responses by activating various Toll-like receptors (TLRs) through binding to a range of lipoproteins and lipopolysaccharides. MTB secretes specific antigens (Rv0577, Rv2660c, Rv3875, Rv3628, Rv2873, and Rv1808) that are recognized by TLR2, leading to dendritic cell maturation and the induction of Th1/Th17 response in tuberculosis immunity and inflammatory reactions. Similar to TLR2, TLR4 recognizes MTB antigens (Rv3478, Rv3417c, Rv0440, Rv0652, Rv0475, Rv1009, and 38 kD glycoprotein) in conjunction with dectin-1, resulting in apoptosis of MTB-infected macrophages and the production of IL-17A. Additionally, TLR9 recognizes MTB’s CpG DNA, promoting IFN-α production and regulating the Th1/Th2 balance. TBK1 TANK-binding kinase 1, TIRAP Toll/interleukin 1 receptor domain-containing adaptor protein, TRAF3 tumor necrosis factor receptor factor 3, IRF7 interferon regulatory factor 7, type I IFN type I interferon, TRIF Toll/interleukin 1 receptor-domain-containing adapter-inducing interferon-β, TAK1 transforming growth factor β activated kinase 1, IKKs inhibitor of nuclear factor κB kinases, NF-κB nuclear factor κB, IFN-γ interferon-γ, TNF-α tumor necrosis factor-α, IL interleukins

In humans, 10 Toll-like receptors (TLRs) have been identified, including TLR1 for bacterial lipoproteins, TLR2 and TLR6 for lipopeptides, lipoteichoic acid, and peptidoglycan, TLR3 for double-stranded RNA, TLR4 for bacterial lipopolysaccharide (LPS), TLR5 for bacterial flagellin, and TLR7 and TLR8 for single-stranded RNA, TLR9 for CpG DNA motifs, and TLR10 for unknown function. Among these TLRs, TLR2, TLR4, TLR8, and TLR9 are the primary participants in MTB recognition on different cells with diverse activation mechanisms, inducing the production of pro-inflammatory cytokines and chemokines (Fig. 3) [103]. Moreover, recent systematic review and meta-analysis suggest that single-nucleotide polymorphism variants within TLR1, TLR2, TLR4, TLR6, and TLR9 correlate with TB susceptibility and defense [104, 105]. Therefore, TLRs play a vital role in connecting innate and adaptive immunity against MTB. Different TLRs recognize and bind with specific PAMPs, leading to unique signaling pathways that mediate the immune responses. The identification of TLRs and the understanding of their roles in MTB defense are of utmost importance in the development of better diagnostic tools and the discovery of new drugs and vaccines against MTB.

TLR4 is the most important member of the TLR protein family and is involved in the recognition of LPS as well as LPS-mediated inflammatory responses. TLR4 recognizes MTB antigens such as Rv3478 (PPE60), Rv3417c (groEL1), Rv0440 (groEL2), Rv0652 (RplL), Rv0475 (HBHA), Rv1009 (RpfB), and 38 kD glycoprotein, which activate and stimulate macrophages, DCs, and Th1 cells to secrete pro-inflammatory cytokines by recruiting Toll/interleukin 1 receptor domain-containing adaptor protein and Toll/interleukin 1 receptor-domain-containing adapter-inducing interferon-β (TRIF)-related adaptors and downstream MyD88 and TRIF-containing TIR domains [103, 106, 107]. These processes activate the NF-κB pathway, resulting in the expression of inflammatory cytokines such as IL-1, IL-6, and TNF-α that ultimately contribute to MTB clearance [108]. Previous studies have shown the significant role of TLR4 in the recognition of MTB. Macrophages from TLR4-deficient mice have reduced ability to secrete TNF-α, and TLR4-deficient mice have similar susceptibility to MTB infection as TLR2-deficient mice [109, 110]. Additionally, a study has indicated that TLR-deficient mice have higher MTB loads in the lungs, spleen, and liver and lower survival rates after infection than wild-type mice [111]. Despite extensive research on the role of TLR4 in the recognition of MTB, there is still heterogeneity in these findings, highlighting the need for further studies to elucidate the sources of these differences and the role of TLR4 in MTB infection [112].

TLR2 is a transmembrane receptor expressed in immune cells and lung epithelial cells. While TLR2 is not crucial for protection during acute MTB infection, it plays an important and multifaceted role in controlling chronic MTB infection [113]. TLR2 commonly forms a heterodimer with TLR1 or TLR6 and recognizes MTB antigens such as Rv0577 (TB27.3), Rv2660c, Rv3875 [early secreted antigen target-6 (ESAT-6)], Rv3628, Rv2873 (MPT83), and Rv1808 (PPE32), leading to active macrophages, NK cells, CD4^+^ T cells, and DCs to produce cytokines to kill MTB or maintain MTB in LTBI phase via a cascade reaction through the MyD88 pathway that upregulates the expression of genes [103]. According to previous studies, TLR2-deficient mice have shown defects in granuloma formation and increased susceptibility to high-dose MTB infection [110, 114]. They also exhibited a disadvantage in controlling chronic MTB infection compared to wild-type mice [114]. Additionally, cell-based assays have demonstrated that high expression of TLR2 is associated with apoptosis of MTB-infected macrophages, suggesting that TLR2-dependent host macrophage apoptosis can expose hidden MTB for killing [115]. In cohort studies, individuals carrying the rs5743708 nucleotide polymorphism in the *TLR2* gene had a higher risk of developing TB compared to the control group [116]. The above findings suggest that TLR2-mediated recognition of MTB can activate macrophages to produce an inflammatory response, defending against MTB infection. However, TLR2 may also play a role in aiding MTB immune evasion, especially through the induction of IL-10 release [117].

In contrast to TLR2 and TLR4, TLR9 is an intracellular recognition receptor that detects MTB’s unmethylated CpG motifs in DNA and activates MyD88-TRAF3 pathway. This leads to the release of type-I interferon (type I IFN) and interferon regulatory factor 7 (IRF7) upregulation, resulting in a bactericidal or antiviral effect [118]. In vitro studies have shown that the recognition of MTB by TLR9 can activate DCs and induce high levels of IL-12 production [119, 120]. Additionally, in vivo experiments have shown that TLR9-deficient mice have significantly higher mortality rates and earlier time of death when infected with high doses of MTB compared to wild-type mice [119]. There is evidence to suggest that TLR9 promotes the maturation of CD8^+^ T cells and their recognition of MTB antigens by inducing the secretion of type I IFN [121]. On the other hand, a cohort study has shown that blocking TLR9 and TLR4 can significantly reduce the number of Tregs and the expression of IL-10 in LTBI individuals, thereby enhancing host killing of MTB, indicating that MTB infection may activate the TLR4 and TLR9 pathways to suppress immune responses in the LTBI population [122].

Taken together, TLRs are crucial in recognizing antigens and activating macrophages, DCs, and other cells involved in MTB innate immunity [123]. Understanding TLRs’ functions can aid in the early diagnosis of LTBI and reduce its conversion to ATB. By regulating specific TLR pathways, researchers can design new preventive and therapeutic measures for MTB control. Studies suggest that TLR2 pathway stimulation enhances protective immunity against MTB [124], while TLR4 receptor defects increase susceptibility to MTB and other pathogens [125]. Furthermore, understanding TLRs in different populations or different stages of MTB infection can guide the differential diagnosis of LTBI.

### Immune evasion of MTB

**Fig. 4:**
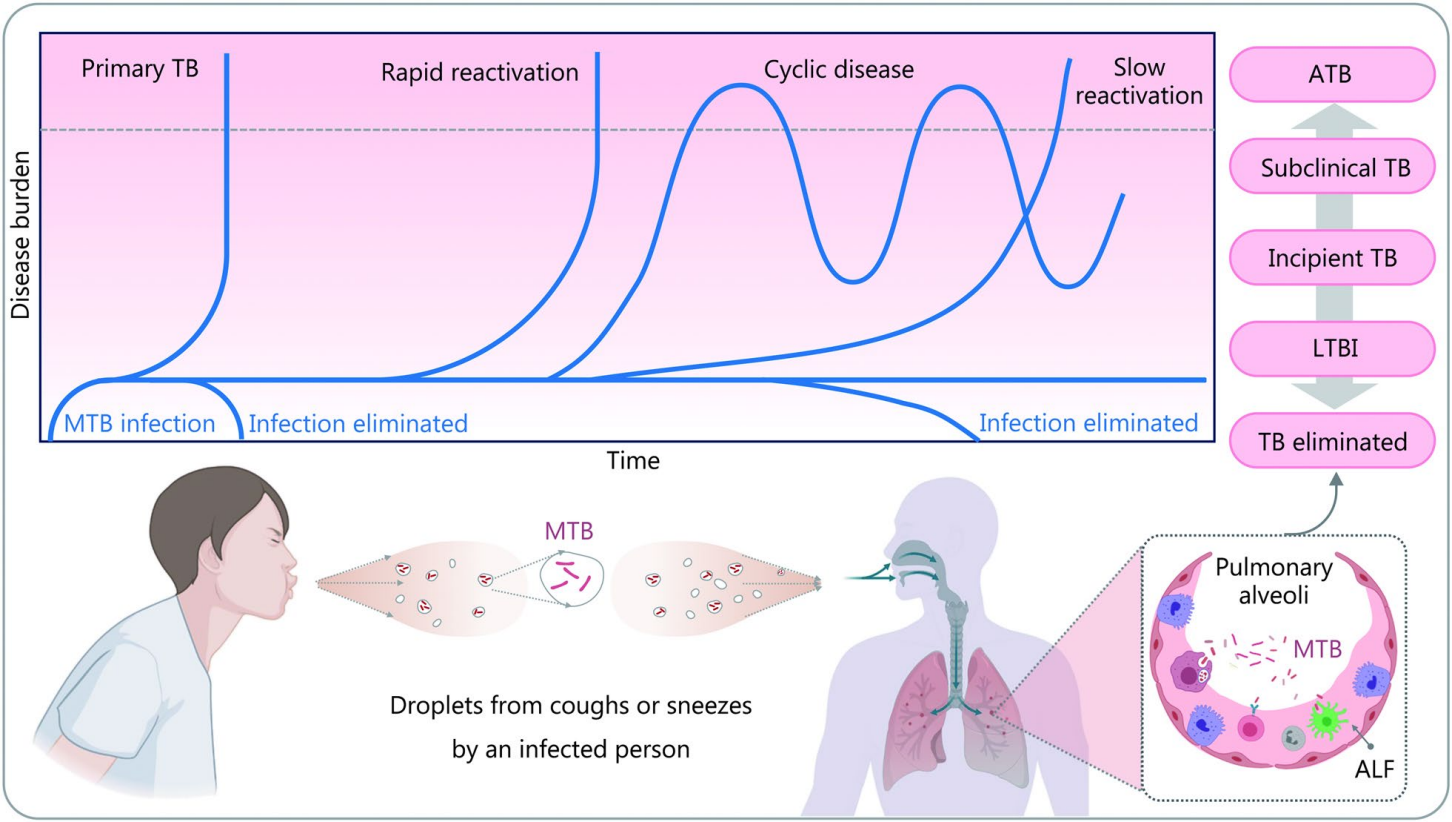
Schematic representation of different outcomes and states after MTB infection of the host. The first outcome is active tuberculosis (ATB), where granulomas rupture allowing MTB to multiply in large numbers and enter the alveoli and surrounding tissues, causing the development of ATB. This condition commonly occurs in individuals with a weakened immune system, such as those with HIV infection or receiving immunosuppressive therapy, or in people with impaired immune function due to other reasons. The second outcome is TB elimination, which occurs when the immune response is sufficient to clear the MTB infection. The third outcome is an intermediate state, where MTB becomes dormant and stops replicating when the host can restrain its virulence or when MTB loads are low, leading to an LTBI, incipient TB, or subclinical TB, that may reactivate when the immune system becomes impaired. The upper part of this figure is a modification of Fig. 1 by Drain et al. [128], 2018. MTB *Mycobacterium tuberculosis*, TB tuberculosis, LTBI latent tuberculosis infection, ALF airway lining fluid

Over thousands of years of evolution, MTB has developed a set of abilities to persist in the host and spread to other individuals despite immune attacks. Whether MTB can complete this infectious cycle depends on a dynamic balance between immunological control and bacterial persistence, which determines its survival [126]. Currently, it has been established that MTB infection presents as a continuum, with ATB and TB elimination on the opposite ends, and LTBI, incipient TB, and subclinical TB in between (Fig. 4) [127–129].

MTB employs various strategies to evade immune attacks and clearance by the host. The first strategy is intracellular parasitism, where MTB survives within host cells, particularly macrophages, by inducing them to secrete type I IFN and inhibiting the production of cytokines like IL-12 and TNF-α [130, 131]. This process, along with ROS suppression, enables MTB to evade direct attacks by the immune system and survive intracellularly. The second strategy is the inhibition of host macrophage activity through the secretion of various molecules by MTB, like triggering receptors expressed on myeloid cells 2, EsxA (Rv3875), and Hsp60 (Rv0440) [132–134]. These molecules interfere with macrophage activation, reducing the effectiveness of host immune responses and prolonging MTB survival within host cells. Finally, MTB can also avoid eradication by inhibiting or delaying the activation of CD4^+^ T cells, which are crucial in mounting an immune response against MTB. Proteins such as groEL2, EsxH, and PE_PGRS47, secreted by MTB, inhibit or delay CD4^+^ T cell activation, providing a window of time for MTB to proliferate unrestricted [135–137].

**Fig. 5:**
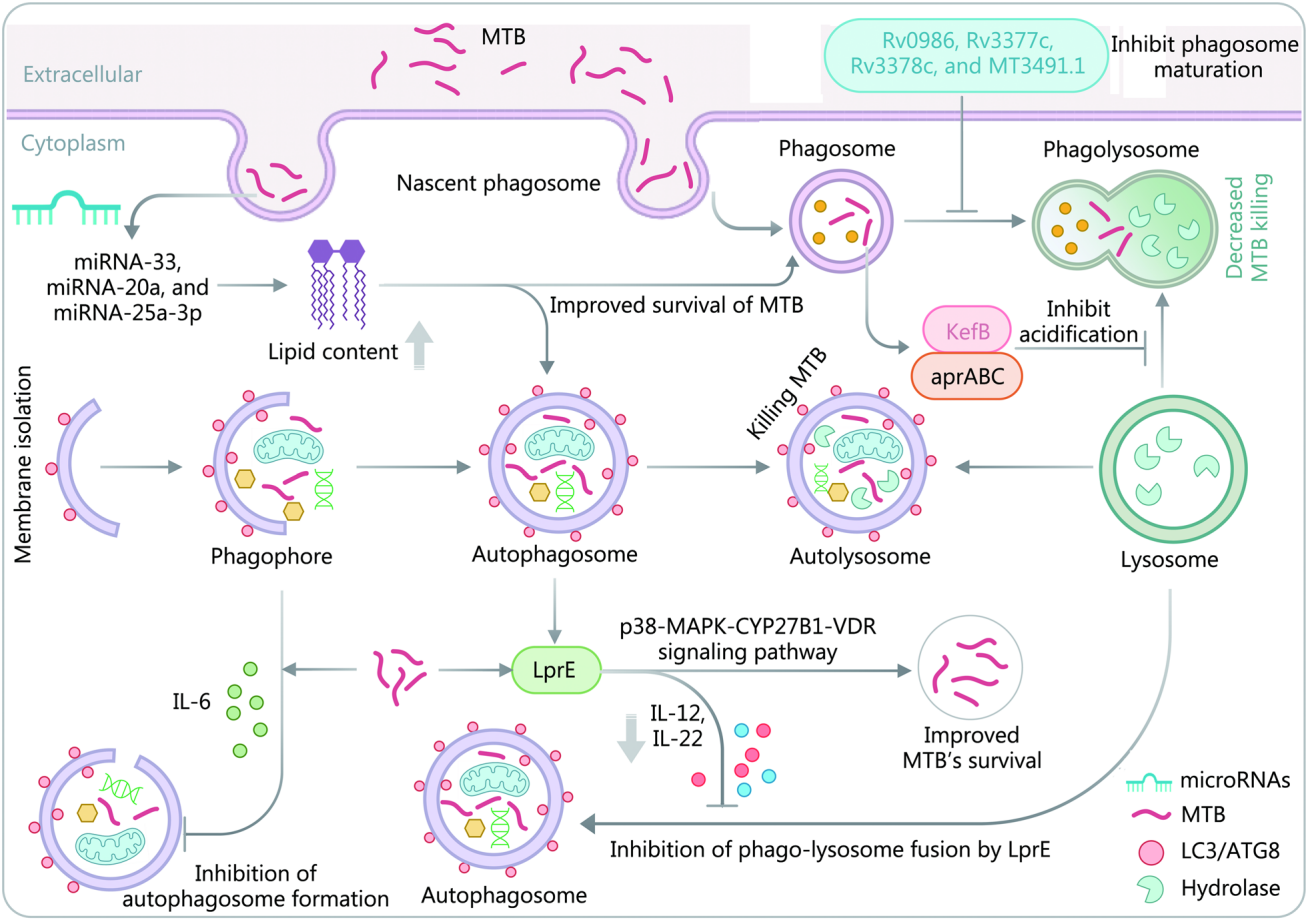
Evasion of autophagic-lysosomal and phagocytic-lysosomal killing by MTB. The bactericidal process of autophagic-lysosomal degradation involves the formation of autophagic precursors that engulf the infected cells to create autophagosomes which then fuse with lysosomes. This results in the hydrolysis of infected cells by lysosomal enzymes. However, in the presence of toxic MTB, the formation of autophagic precursors is inhibited through the regulation of cytokine production. Additionally, MTB’s lipoproteins LprE can delay the fusion of phagocytic lysosomes by regulating cytokines production, leading to the evasion of phagocytic-lysosomal killing. The phagocytic process involves the engulfment of MTB vesicles by lysosomes containing acid hydrolases that can kill MTB. MTB evades phagocytic-lysosomal killing in various ways. MTB *Mycobacterium tuberculosis*, IL interleukin, KefB a potassium/proton antiporter in MTB (Rv3236c), aprABC an MTB complex-specific locus, MAPK mitogen-activated protein kinase, CYP27B1 1 alpha-hydroxylase, VDR vitamin D receptor, LC microtubule-associated protein light chain, ATG8 autophagy associated proteins 8

The immune evasion mechanisms employed by MTB play a central role in the long-term persistence of TB in the host. To better understand these strategies, recent studies have explored various mechanisms like phagocytosis, autophagy, and apoptosis utilized by MTB in the host (Fig. 5) [138–140]. In addition to studying the mechanisms involved in MTB immune evasion, researchers have explored the energy source and survival status of MTB during latent infection as another approach for discovering biomarkers for differential diagnosis of LTBI.

#### Phagocytosis

MTB has evolved several mechanisms to evade the host immune response and inhibit phagosome-lysosome fusion, a process that is crucial for killing intracellular pathogens. Upon being engulfed by host phagocytic cells, MTB utilizes various mechanisms to inhibit phagosome-lysosome fusion and evade the host’s immune response to maintain its survival and replication within infected cells [141]. To prevent phagosomes from acidifying, MTB can inhibit access to the V-ATPase enzyme [142–144], which is required for acidification, as well as produce KefB to inhibit phagosomal acidification [145]. Additionally, MTB can escape from host cells containing phagolysosomes into non-apoptotic cells, a process meditated by secretion of ESAT-6 secretion system 1 (ESX-1) [146, 147], which allows it to maintain replication or enter into a latent state in the host [148]. MTB can also adapt to the acidic environment inside phagosomes by manipulating the phoPR (possible two component system response sensor kinase membrane associated PhoR and transcriptional positive regulator PhoP) operon to regulate MTB-specific locus aprABC (an MTB complex-specific locus), which in turn initiates cell wall lipid synthesis [149]. Interestingly, a previous study has found that MTB mutants with mutations in Rv0986, Rv3377c, Rv3378c, and MT3491.1 antigens are more susceptible to capture and subsequent elimination by phagosomes with lower pH compared to wild-type MTB, suggesting that these MTB antigens may play a crucial role in inhibiting phagosome maturation and acidification, and may act as key regulatory factors for sustained MTB survival within macrophages [150]. Despite these various strategies employed by MTB to evade or inhibit phagosomes, our understanding of these mechanisms is still incomplete, and further research is needed to gain a deeper insight into the survival and proliferation strategy of MTB and its associated immune mechanisms. Such insights could help develop new therapeutic approaches to combat MTB and reduce the incidence of TB.

#### Autophagy

In the context of MTB infection, autophagy can eliminate the pathogen by degrading it within autophagosomes that eventually fuse with lysosomes [151, 152]. However, MTB has evolved several mechanisms to suppress autophagy in host cells, thereby compromising the ability of the host to clear the pathogen. MTB can suppress autophagy by modulating various immune signaling pathways (Fig. 5). For instance, it can inhibit autophagy by regulating IL-6 and LprE-binding protein levels, which directly inhibit autophagy and promote MTB survival within the host [153]. Additionally, MTB can inhibit autophagy by regulating various microRNAs such as miR-33, miR-20a, and miR-25a-3p, which lead to increased lipid content and improved survival of MTB within the host [154–156]. Lipid bodies serve as an essential nutrient source for MTB, increasing its survival capacity within the host. LprE lipoprotein can inhibit vitamin D_3_ expression via the TLR2-dependent p38-MAPK-CYP27B1-VDR signaling pathway, thereby promoting MTB survival within the host and compromising the host’s ability to kill the pathogen [157]. In contrast, augmented vitamin D_3_ levels can kill MTB within the host. Thus, MTB uses several mechanisms to suppress autophagy and evade the host immune response. Identifying and targeting these mechanisms could provide new therapeutic avenues for controlling and preventing TB. As a result, studies into understanding these mechanisms can provide insights into discovering new therapeutic targets for developing effective TB treatment strategies.

#### Apoptosis

Apoptosis is a self-protective mechanism against dangerous pathogens, aiding in pathogen clearance. However, MTB has evolved several mechanisms to manipulate cell death [158]. MTB’s anti-apoptotic antigens, such as protein tyrosine kinase transcriptional regulatory protein (PtPA, Rv2232), NADH-ubiquinone oxidoreductase chain G (NuoG, Rv3151), and alternative RNA polymerase sigma-E factor (SigH, Rv3223c), competitively bind to the really interesting new gene (*RING*) domains of tripartite motif proteins, inhibiting the apoptosis pathway and promoting MTB’s survival inside the host [159–162]. Similarly, MTB can suppress cell death by inhibiting Fas receptor (FasR) expression or inducing IL-10 production [163]. Different MTB strains of varying virulence can induce apoptosis or necrosis for evasion of clearance and intracellular replication. For instance, highly virulent MTB H37Rv selectively causes macrophage necrosis, while attenuated MTB H37Ra tends to induce apoptosis [164]. Host cell response to MTB invasion results in both apoptosis and necrosis, and MTB of distinct virulence may generate diverse cell death types. Thus, the investigation into the mechanisms of different cell death induction types by MTB is essential to understanding LTBI. Exploration of the strategies and mechanisms involved in MTB’s survival and evasion within the host is key to understanding the mechanisms underlying LTBI. By deciphering MTB’s ability to persist and evade extensive infection, it may provide new biomarkers for differential diagnosis of LTBI.

### Energy sources and regulation mechanisms of MTB during latency and granuloma formation

Studies have revealed that fatty acids and cholesterol serve as the primary energy source for MTB during the latency and granuloma formation stages within the host [165, 166]. However, the metabolite of fatty acids and cholesterol, propionyl-CoA, demonstrates toxicity to MTB [167]. The detoxification of propionyl-CoA relies on the activity of the methylcitrate cycle, the methylmalonyl pathway, or the incorporation of propionyl-CoA into methyl-branched lipids in the cell wall [166]. MTB must utilize gene-encoded proteins, such as membrane-associated phospholipase C 1 (plcA, Rv2351c), plcB (Rv2350c), plcC (Rv2349c), plcD (Rv1755c), isocitrate lyases (ICLs) like Rv0467, Rv1915, and Rv1916, and malate synthases (MS) like Rv1837c, to overcome this toxicity and obtain the necessary energy during the latency and granuloma formation periods [168–170]. This process is referred to as acetate metabolism, which exhibits similarities with microbial and plant metabolic processes [171]. Research has shown that deletion of ICL significantly hinders MTB replication and growth, and knockout of Rv3671c leads to a decrease in acid resistance in MTB [172, 173]. Due to a lack of oxygen and nutrients within TB granulomas, anaerobic glycolysis occurs, ATP levels decline, and replicating MTB converts into non-replicating states, allowing for long-term survival within the environment. However, this survival may require ICLs regulation [174]. Therefore, energy sources are an essential factor for MTB survival and replication inside the host, and regulatory mechanisms require further investigation, indicating that these MTB’s antigens may be promising biomarkers for LTBI diagnosis.

### The role of epigenetics and gene regulation in MTB infection

Epigenetic regulation plays a vital role in MTB adaptation and survival within the host. DNA methylation, histone modifications, miRNA regulation, and other mechanisms can influence or alter gene expression, allowing MTB to adapt quickly to the host environment and evade immune attacks [175, 176]. MTB has been found to regulate DNA methylation to accelerate cell senescence and inhibit an immune response, promoting survival within the host by controlling inflammatory cytokine response and cell apoptosis [177, 178]. miRNA expression also plays an essential role in MTB infection, where thousands of miRNAs can regulate transcription after mRNA [179, 180]. For instance, miR-33a/b can increase lipid levels and provide an energy source for MTB by inhibiting cholesterol biosynthesis genes and regulating fatty acid oxidation [181]. Additionally, miRNAs such as miR-27a-5p, miR-33, and miR-125-5p can suppress autophagy, reduce macrophage-killing capacity, and control TNF receptor-associated factor 6 to lower immune responses, thereby enhancing MTB survival [3, 182, 183]. Moreover, miR-29a-3p, the most highly expressed miRNA in latent TB patients, can suppress the host’s immune response, decrease IFN-γ level, and escape macrophage phagocytosis through cell apoptosis [184, 185]. Recently, a meta-analysis of 21 studies identified miR-29, miR-31, miR-125b, miR-146a, and miR-155 as potential biomarkers for ATB diagnosis [186]. The overall sensitivity, specificity, and diagnostic odds ratio (*DOR*) for these biomarkers in ATB diagnosis were 87.9% (81.7–92.2%), 81.2% (74.5–86.5%), and 43.1 (20.3–91.3), respectively [186]. These findings highlight that the differential expression of miRNA during MTB infection may provide insights into developing novel biomarkers to distinguish between ATB and LTBI. The unique miRNA signatures of ATB and LTBI can differentiate between the two states, and these signatures may serve as potential biomarkers for early and accurate diagnoses.

## Status and challenges of discriminating diagnosis of LTBI

In recent years, significant progress has been made in the research on LTBI. Despite these advances, the diagnostic difficulties of LTBI remain, including issues such as cost, detection time, sensitivity, and specificity [68]. Although some new biomarkers have been proposed for the discrimination diagnosis of LTBI, there are practical implementation issues that need to be addressed. Currently, the most widely used methods for diagnosing LTBI are TST and IGRA. TST is a traditional and established diagnostic method with affordable, simple to perform, and requirement of minimal laboratory equipment [13]; however, its results can be affected by BCG vaccination and non-tuberculous mycobacterial infections. On the other hand, IGRA is a new detection method that can distinguish between BCG vaccination and MTB infection, but its results are influenced by the host’s condition and have lower sensitivity for TB patients and immunosuppressed patients. Both methods cannot differentiate between LTBI and ATB populations.

One major challenge or concern in the differential diagnosis of LTBI is the selection of LTBI criteria. The fundamental attributes of individuals with LTBI, such as comorbidities, immune status, and genetic factors, have an impact on their immune response and disease outcomes. Given the heterogeneity of LTBI, it is imperative to carefully select study participants based on standardized inclusion and exclusion criteria while considering these fundamental factors during the data analysis phase. This rigorous approach ensures the reliability and applicability of research findings to the specific LTBI population under investigation.

In the following section, we will review the diagnostic use of TST and IGRA in distinguishing LTBI and the obstacles they face.

### TSTs

#### Purified protein derivative (PPD) test

The PPD test, commonly known as the tuberculin test, is a type of intradermal test used to diagnose type IV hypersensitivity reactions resulting from infection with MTB [13]. Although the test is highly sensitive, some factors impact its accuracy, including BCG vaccination, non-tuberculous mycobacterial infection, and malnutrition, leading to false positive or false negative results [187]. In addition, the diagnostic accuracy of the test may vary due to differences in the tuberculin extract used, leading to issues with consistency. Nonetheless, TST is a low-cost, widely available, and well-established test that has been recommended for use in medium or underdeveloped countries [188].

#### Newly developed TSTs

Despite the limitations of the tuberculin test, several new skin tests have been developed in recent years, such as C-TB, Diaskintest, and EC-Test (Table 1) [189–194]. Although these tests have overcome some of the limitations of the tuberculin test to some extent, they still have issues with limited applicability, diagnostic accuracy, cost, and technical difficulty. C-TB is a skin test developed based on the ESAT-6 and culture filtrate protein-10 (CFP-10) antigens secreted by MTB, which has lower sensitivity than the tuberculin test but has high specificity in healthy controls vaccinated with BCG [189]. Diaskintest is a skin test based on a complex of CFP-10/ESAT6 recombinant proteins developed in Russia. Its sensitivity is similar to TST, and it is low in cost and easy to operate, with results similar to those of IGRAs. It is recommended by the WHO for implementation in countries with limited resources and widespread BCG vaccination [195, 196]. The EC-Test is a TB detection test kit based on ESAT-6 and CFP-10 antigens, which have high sensitivity and specificity and have been validated in clinical trials [197–199]. While these new skin tests have the advantages of being relatively low in cost, easy to operate, and safe, they still need to be used with special attention to their applicability and cannot completely differentiate between ATB and LTBI. Further large-scale trials are necessary to verify their effectiveness.

**Table 1:** Current TST methods used for LTBI diagnosis

Characteristics	PPD	C-TB	Diaskintest	EC-test
Time	> 100 years	2009	2010	2020
Type of reaction	DTH	DTH	DTH	DTH
Number of visits	2	2	2	2
Type of antigen	PPD	ESAT-6 and CFP-10	ESAT-6 and CFP-10	ESAT-6 and CFP-10
Outcome measures	Millimeters of induration	Millimeters of induration	Millimeters of induration	Millimeters of induration
Sensitivity	77% [190] 84% [191]	73.9% [189] 74.52% [192]	86% [193] 68% [193] 91.18 [192]	90.85% [194] 86.06% [192]
Specificity	97% (without BCG vaccination) and 59% (with BCG vaccination) [190] 100% (without BCG vaccination) and 79% (with BCG vaccination) [191]	97.85% [192]	98% [193]	89.83% [194]
Interpretation	Subjective	Subjective	Subjective	Subjective
False positive rate in immunosuppressed or BCG vaccinated population	High	Low	Low	Low
Distinguish between LTBI and ATB	No	No	No	No

*ATB* active tuberculosis, *BCG* Bacillus Calmette–Guérin, *CFP-10* culture filtrate protein-10, *DTH* delayed type hypersensitivity, *ESAT-6* early secreted antigen target-6, *LTBI* latent tuberculosis infection, *TST* tuberculin skin test, *PPD* purified protein derivative, *C-TB* a novel skin test based on ESAT-6 and CFP-10 proteins, *EC* recombinant *Mycobacterium tuberculosis* fusion protein of ESAT-6 and CFP-10

### IGRA

Given the limitations of traditional TSTs, a new diagnostic method has been developed, called IGRA. The principle of the method involves stimulating whole blood cells with MTB antigens in vitro, and determining whether or not the individual has been infected with MTB by measuring the amount of IFN-γ produced after stimulation or the number of CD4^+^/CD8^+^ T cells that release IFN-γ. Currently, there are 3 IGRA tests recommended by the WHO for detecting TB, namely the T-SPOT.TB spot test (Oxford Immunotec, UK), QuantiFERON-TB Gold Plus (QFT-Plus, Qiagen, USA), and Wantai TB-IGRA (Wantai, China). In addition, several assays are either being launched or currently in development, such as QIAreach™ QuantiFERON-TB (QIAreach QFT) (Qiagen, USA), Standard E TB-Feron (SD Biosensor, Korea), LIOFeron TB/LTBI (LIONEX Diagnostics & Therapeutics GmbH, Germany), VIDAS™ TB-IGRA (bioMérieux, France) and AdvanSure™ TB-IGRA enzyme-linked immunosorbent assay (ELISA) (LG Life Sciences, Seoul, Korea). Currently, three other IGRAs are under development, including ichroma™ IGRA-TB (Boditech Med Inc., Korea), T-Track^®^ TB (Mikrogen GmbH, Neuried, Germany), and interferon protein-10 (IP-10) IGRA ELISA/lateral flow (rBioPharm, Germany) [2]. It should be noted that since the QuantiFERON-TB Gold In-Tube (QFT-GIT, Qiagen GmbH, Germany) has been replaced by QFT-Plus, and 5 commercial assay kits have been introduced in our previous study [13], this review will focus on the introduction and comparison of other newly launched or under development IGRAs, including AdvanSure™ TB-IGRA ELISA, Wantai TB-IGRA, Standard E TB-Feron (TBF), QIAreach QFT, ichroma™ IGRA-TB, VIDAS™ TB-IGRA, and T-Track^®^ TB (Table 2).

**Table 2:** Latest IGRAs methods used for LTBI diagnosis

Parameters	T-SPOT.TB	QFT-plus	LIAISON QFT-plus	AdvanSure™ TB-IGRA	Wantai TB-IGRA	Standard E TB-feron	QIAreach QFT	ichroma™ IGRA-TB	VIDAS™ TB-IGRA	T-Track^®^ TB
Manufacturer	Oxford, Immunotec, United Kingdom	Hilden, Germany	DiaSorin S.P.A., Italy	LG Life Sciences, Korea	Wantai, China	SD Biosensor, Korea	Hilden, Germany	Boditech Med Inc., Korea	bioMérieux SA—Marcy-l’Étoile, France	Mikrogen GmbH, Neuried, Germany
Sample	PBMC	Whole blood	Whole blood	Whole blood	Whole blood	Whole blood	Whole blood	Whole blood	Whole blood	Whole blood
Type of reaction	T cell immune response	T cell immune response	T cell immune response	T cell immune response	T cell immune response	T cell immune response	T cell immune response	T cell immune response	T cell immune response	T cell immune response
Number of visits	1	1	1	1	1	1	1	1	1	1
Type of antigen	Panel A: ESAT-6 Panel B: CFP-10	ESAT-6, CFP-10	ESAT-6, CFP-10	ESAT-6, CFP-10	ESAT-6, CFP-10	ESAT-6, CFP-10, and TB7.7 recombinant proteins	A peptide cocktail simulating the proteins ESAT-6 and CFP-10	ESAT-6, CFP-10	ESAT-6, CFP-10	ESAT-6, CFP-10
Tubes	1 tube	Nil, TB1, TB2, and Mitogen tubes	PC, TB-A, TB-B, and NC	Nil, TB, and Mitogen tubes	Nil, TB, and Mitogen tubes	Nil, TB, and Mitogen tubes	Blood collection tube, and processing tube	Nil, TB, and Mitogen tubes	Nil, TB, and Mitogen tubes	Nil, TB, and Mitogen tubes
Technology platform	ELISPOT	ELISA	CLIA	CLIA	ELISA	ELISA	LFIA	FIA	ELISA	RT-qPCR
Results	The number of cells releasing IFN-γ	The amount of IFN-γ released by CD4/CD8 T cells	The amount of IFN-γ released by CD4/CD8 T cells	The amount of IFN-γ released by CD4/CD8 T cells	The amount of IFN-γ released by CD4/CD8 T cells	The amount of IFN-γ released by CD4/CD8 T cells	The amount of IFN-γ released by CD4/CD8 T cells	The amount of IFN-γ released by CD4/CD8 T cells	The amount of IFN-γ released by CD4/CD8 T cells	Levels of *FNG* and *CXCL10* mRNA changes
Sensitivity	83% [13]	91.4% [200]	78.6% [201]	98.48% agreement with QFT [202]	86.4% [203]	88% [204]	100.0% [205], 96.5% agreement with QFT PLUS [206]	95.76% [207]	97.5% [208]	94.9% [209]
Specificity	83.1% [13]	97.8% [200]	94.7% [201]	97.95% agreement with QFT [202]	85.9% [203]	95% [204]	97.6% [205], 94.2% agreement with QFT PLUS [206]	88% [207]	97.6% [https://www.biomerieux-diagnostics.com/vidasr-tb-igra]	93.8% [209]
False positive rate	Low	Low	Low	Low	Low	Low	Low	Low	Low	Low
Distinguish between LTBI and ATB	No	No	No	No	No	No	No	No	No	No

*ELISPOT* enzyme-linked immunospot assay, *ELISA* enzyme-linked immunosorbent assay, *CLIA* chemiluminescent immunoassay, *IFNG* interferon-γ gene, *LFIA* lateral flow immunoassay, *FIA* fluorescent immunoassay, *T.SPOT* T cell spot test, *QFT* QuantiFERON-TB-Gold-In-Tube, *IGRA* interferon gamma release assay, *QIAreach* registered trademark name for a kit for the detection of *Mycobacterium tuberculosis* from Qiagen (a commercial company in Germany), *VIDAS* a Registered trademark name of bioMérieux, *PBMC* peripheral blood mononuclear cell, *ESAT-6* early secretary antigenic target-6, *CFP-10* culture filtrate protein-10, *PC* positive control, *TB-A* tuberculosis-A, *TB-B* tuberculosis-B, *NC* negative control, *RT-qPCR* reverse transcription-quantitative real-time polymerase chain reaction, *IFN-γ* interferon-γ, *CXCL* chemokine ligand

#### AdvanSure™ TB IGRA

AdvanSure™ TB-IGRA ELISA is a novel IGRA test technology developed by SD Biosensor in Korea, which is based on an automated chemiluminescence immunoassay system and utilizes 3 testing tubes [a negative control tube (Nil), a positive control tube (Mitogen), and a TB antigen tube with ESAT-6 and CFP-10 antigens] for diagnosis. A comparative study evaluated the LTBI discriminatory diagnostic performance of AdvanSure™ TB-IGRA and compared it with the diagnostic efficacy of QFT-GIT. The results showed that the repeatability and reproducibility of this technology were 4.86–7.00% and 6.36–7.88% coefficient of variation (CV), respectively, and its diagnostic performance was 99.1%, consistent with QFT-GIT [202]. Additionally, as one of the IGRA technologies, AdvanSure™ TB-IGRA can efficiently diagnose TB and has practical value in the rapid screening of TB infection and diagnosis of infected individuals. However, it should be noted that further clinical trials and validation of AdvanSure™ TB-IGRA are required to ensure the reliability of its diagnostic accuracy and safety.

#### Wantai TB-IGRA

Wantai TB-IGRA, developed by Beijing Wantai, is a new commercial IGRA and one of the three IGRA kits recommended by the WHO. The kit is based on the ELISA method and includes a negative control tube (Nil), a positive control tube (Mitogen), and a TB antigen tube with ESAT-6 and CFP-10 antigens. In previous studies, Wantai TB-IGRA showed no significant difference in sensitivity compared to QFT-GIT and T-SPOT [210, 211]. In addition, Wantai TB-IGRA is relatively low-cost. However, it is worth noting that Wantai TB-IGRA is a new diagnostic method that has shown some advantages over traditional methods such as the TST but also has certain limitations. Although Wantai TB-IGRA has shown sensitivity comparable to QFT-GIT and T-SPOT in current comparative studies, there are still challenges in the diagnosis and differential diagnosis of TB, especially in distinguishing between LTBI and ATB. Moreover, although Wantai TB-IGRA is low-cost, factors such as cross-reactivity need to be taken into consideration.

#### Standard E TB-Feron (TBF)

TBF is an IGRA developed in Korea, whose principle is similar to that of QFT-GIT, but with better time and cost efficiency. The main difference is that the antigen tube in TBF contains the whole recombinant protein of ESAT-6, CFP-10, and TB7.7, while QFT-GIT uses synthetic peptide antigens of ESAT-6, CFP-10, and TB7.7 [212]. A study with 335 participants compared the heterogeneity and diagnostic efficacy of TBF and QFT-Plus. The results showed that the sensitivity and specificity of TBF were 88% and 95%, respectively, and the positive consistency rate between TBF and QFT-Plus was 94.0% [204]. It should be noted that although TBF has advantages in consistency and time cost, it is not superior to QFT-Plus and T-SPOT in sensitivity and specificity and cannot directly distinguish LTBI from ATB. Therefore, when selecting IGRA as a diagnostic tool, a comprehensive analysis should be conducted with full evaluation and consideration of clinical practice and laboratory test results.

#### QIAreach QFT

We know that ELISA-based IGRAs require complex steps and time-consuming, and require laboratory infrastructure and well-trained technical personnel to complete [213]. Therefore, a new semi-automated lateral flow immunoassay has been developed, which is the QIAreach QFT developed by Qiagen in Germany. Its antigen tube is the same as the TB2 antigen tube in QFT-PLUS, which can stimulate CD4^+^ and CD8^+^ T cells to produce interferon. The difference lies in its coupling with dilution buffer and nanoparticles for detection. Nevertheless, the overall operation is still simple, with low technical and detection environment requirements, and requires relatively small amounts of blood [214, 215]. A comparative study showed that in a population of 41 individuals with pulmonary TB and 42 healthy or low TB risk individuals, the sensitivity and specificity of QIAreach QFT compared to QFT-PLUS were 100.0% (41/41) and 97.6% (41/42), with an overall consistency of 98.8% [205]. In another comparative study, the sensitivity of QIAreach QFT in detecting TB infection in the treatment group and the non-treatment group were 93.7% and 95.1%, respectively, with a specificity of 97.7% and an overall consistency with QFT-Plus of 95.7% [214]. This result is consistent with the results of a recent study [206]. Although QIAreach QFT has the advantages of being easy to operate, requiring low technical facilities and detection environment, and requiring relatively small amounts of blood, this technique still requires further research to accurately evaluate its performance in different environments and study populations, including patients with reduced immune function, HIV-infected individuals, and children.

#### ichroma™ IGRA-TB

The ichroma™ IGRA-TB is an automated diagnostic technology developed by Boditech Med Inc. in Korea, which is based on the fluorescence lateral flow immunoassay (fluorescence lateral flow immunoassay) technology [216]. It is a new point-of-care-testing diagnostic platform, including a set of test antibodies, a buffer solution, and a mobile device (ichroma™ II). The sample and test antibodies only need to incubate for 15 min, and the test process can be completed within 20 min. Previous study has shown that in distinguishing between healthy individuals and TB patients, the area under the receiver operating characteristic curve of ichroma™ IGRA-TB was 0.9706, and the consistency of the detection results with QFT-GIT reached 95.2%, with a strong positive correlation between IFN-γ values detected by the two methods [207]. In addition, another study compared the diagnostic performance of ichroma™ IGRA-TB and QFT-Plus in patients with immune-mediated inflammatory diseases (IMID) for LTBI. In the IMID population, ichroma™ IGRA-TB and QFT-Plus detected 11 (7.6%) and 20 (13.8%) LTBI patients, respectively, with an overall consistency of 91.0% between the two methods [217]. This test technology has the advantages of low cost and ease of use [217]. However, like other IGRA technologies, ichroma™ IGRA-TB cannot distinguish ATB from LTBI. It is worth noting that although ichroma™ IGRA-TB has high sensitivity and specificity in diagnosing LTBI, its clinical value still needs to be confirmed in larger samples and multicenter data. When selecting IGRA technology as a diagnostic tool, we also need to consider the specific situation and actual conditions of different laboratories.

#### VIDAS™ TB-IGRA

VIDAS™ TB-IGRA is a new fully automated method for detecting MTB infection developed by bioMérieux in France. Similar to other TB-IGRA methods, VIDAS™ TB-IGRA also uses ESAT-6 and CFP-10 antigens. Compared to existing TB-IGRA detection schemes, the detection process of VIDAS™ TB-IGRA has been fully automated, and only one tube of whole blood sample is required without the need for manual preparation. In addition to full automation, VIDAS™ TB-IGRA also exhibits strong clinical performance. The VIDAS™ TB-IGRA test technology requires 3 different tubes: a negative control tube (Nil), a positive control tube (Mitogen), and a TB antigen tube. Previous studies have evaluated the immunogenicity of the core component AG of VIDAS™ TB-IGRA. The results showed that AG (MTB antigen) could induce CD4^+^ and CD8^+^ T cells to produce IFN-γ response in LTBI and ATB patients [208], similar to QFT-Plus [218]. In addition, clinical trials in populations from different regions have shown that, compared to existing detection methods, VIDAS™ TB-IGRA has better sensitivity in ATB patients (97.0% vs. 80.6%), high specificity (97.6%) in populations with very low risk of TB infection, and strong consistency with the comparative detection method in populations with mixed risks of TB infection [208, 219]. Despite the strong clinical performance and advantages of full automation demonstrated by VIDAS™ TB-IGRA, more clinical data and research are still needed to demonstrate its value and accuracy in different populations.

#### *T-Track*^*®*^* TB*

T-Track^®^ TB is a new in vitro diagnostic technology developed and manufactured by Mikrogen GmbH in Germany for the detection of MTB infection [2]. It comprehensively evaluates the relative mRNA levels of *IFNG* and *CXCL10* in specific restimulated and unstimulated whole blood samples using reverse transcription-quantitative real-time polymerase chain reaction technology. T-Track^®^ TB includes a negative control tube (Nil), a positive control tube (Mitogen), and a TB antigen tube with the TB antigen being a recombinant ESAT-6/CFP-10 heterodimer protein produced by BL21 (DE3) *Escherichia coli*. A recent case–control study compared the performance of T-Track^®^ TB and QFT-Plus in diagnosing ATB [209]. The study included a total of 541 subjects (including 273 ATB patients and 268 uninfected controls) and tested them according to the respective instructions. The results showed that the sensitivity and specificity of T-Track^®^ TB in diagnosing ATB were 94.9% and 93.8%, respectively, while the sensitivity of QFT-Plus ELISA was 84.3%. The sensitivity of T-Track^®^ TB was significantly higher than that of QFT-Plus (*P* < 0.001). The overall consistency between the two test methods for detecting ATB was 87.9% [209]. It should be noted that this study has limited research on the ability to distinguish LTBI from ATB, and more research is needed to verify its accuracy. Like other TB-IGRA methods, T-Track^®^ TB cannot fully distinguish LTBI from ATB. If it is necessary to accurately locate the TB infection status, we need to combine population epidemiology, clinical manifestations, imaging examinations, and other relevant tests to make comprehensive judgments. Overall, as a new in vitro diagnostic infection detection technology, T-Track^®^ TB has many potential advantages, such as high sensitivity, high specificity, whole blood testing, and short operation time. However, its practical application still requires more large-scale and multicenter studies for verification.

## Application of ML in discriminating diagnosis of LTBI

Although ML has been applied to the diagnosis of ATB [220, 221], differentiation of non-tuberculous mycobacterial lung disease and pulmonary TB [222], the discovery of TB drugs [223, 224], discrimination of drug susceptibility and drug-resistant TB [225], and precise detection of smear-positive/negative pulmonary TB [226], its application in discriminating diagnosis of ATB and LTBI is relatively rare. The main reason is that there are fewer data sources available for LTBI patients. The primary data sources used by ML include medical image data, biomarker data, and clinical information data. However, LTBI patients have no clinical symptoms or imaging features, which makes it impossible to use medical image data and clinical information data for discriminating diagnoses of LTBI. Therefore, the exploration and application of ML in distinguishing diagnosis of LTBI can only be based on biomarker data. Currently, biomarker data mainly comes from various omics data, including transcriptomics, proteomics, metabolomics, etc. Among them, the biomarkers from transcriptomics and proteomics are studied the most. Based on the above objective facts, we will briefly review the concept of ML and its common algorithms and focus on applying ML methods based on transcriptomics and proteomics technologies in discriminating diagnosis of TB latent infection.

### ML and common algorithms

**Fig. 6:**
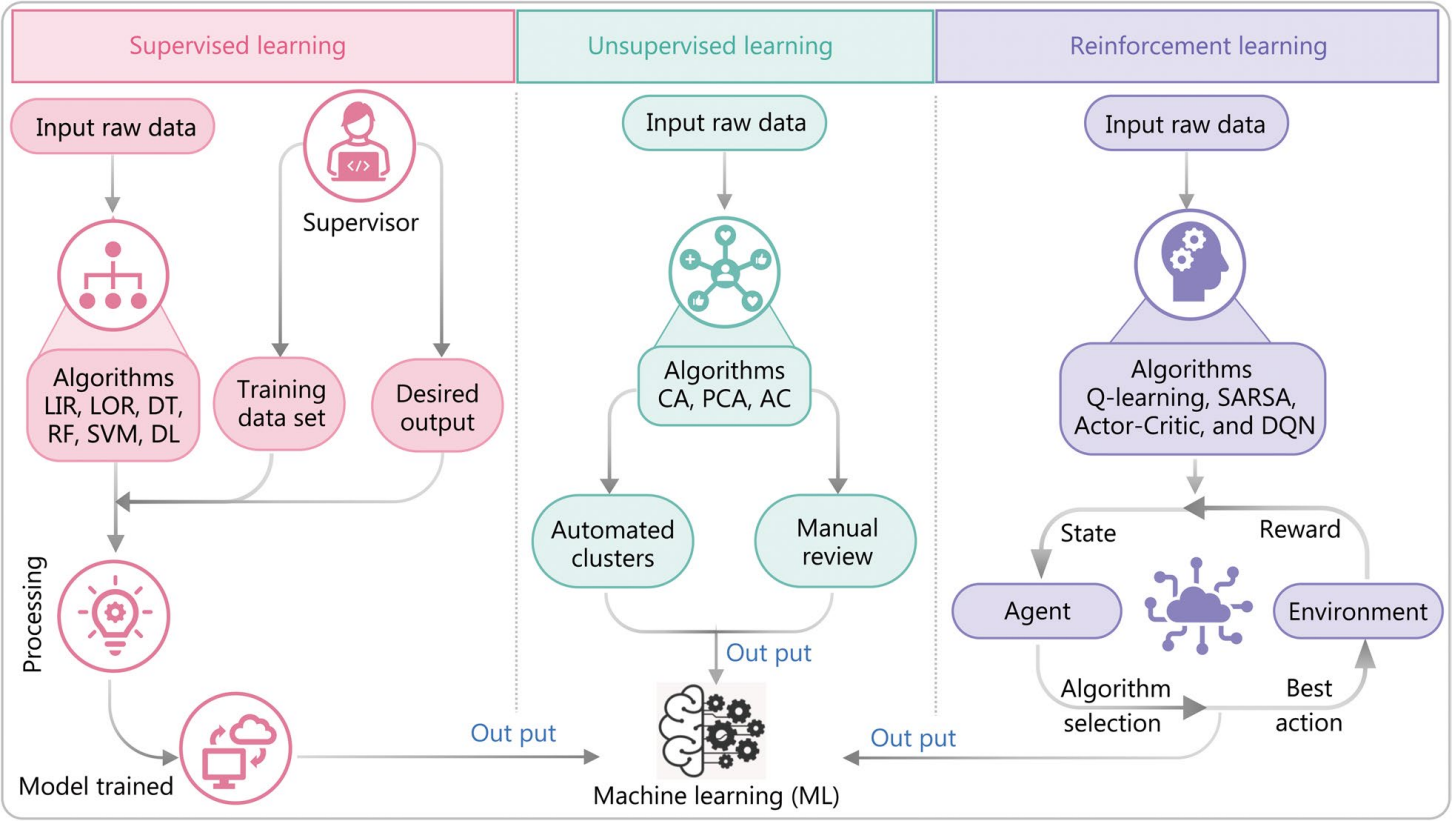
Schematic representation of machine learning classification. AC autoencoder, CA cluster analysis, DL deep learning, DQN Deep Q-Network, DT decision tree, LIR linear regression, LOR logistic regression, PCA principal component analysis, RF random forest, SARSA State-Action-Reward-State-Action, SVM support vector machine

ML is a technique that uses algorithms and models to automatically extract patterns from input data for prediction and decision-making purposes [227]. With the advent of big data, ML has been widely applied in various fields [228, 229]. In the field of TB, ML is also widely used for medical image analysis, drug discovery, disease diagnosis, and treatment [220, 223]. The working mechanism of ML is significantly different from that of traditional computer programs. Traditional computer programs require predefining the logic and rules of the program and then processing the specified input data to obtain the results. In contrast, ML automatically learns patterns and features from the data through training and generates prediction and decision-making models, enabling the prediction and classification of new data [230]. ML can be classified into supervised learning, unsupervised learning, and reinforcement learning based on the learning methods (Fig. 6) [231–233].

One of the most common methods of ML is supervised learning, which learns patterns and models from input–output data with labeled training data. Common supervised learning algorithms include linear regression, logistic regression, decision tree, random forest, support vector machine, and deep learning. In contrast, unsupervised learning infers structures and patterns from unlabeled data. Algorithms for unsupervised learning search for patterns and rules in the data, which require more effort than the labeled data used for supervised learning [234]. Common unsupervised learning algorithms include cluster analysis, principal component analysis (PCA), and autoencoder. Reinforcement learning is a type of ML that learns the optimal decision-making strategy through interacting with the environment. It involves modeling dynamic systems and updating the policy model based on its state, action, and reward. Popular reinforcement learning algorithms include Q-learning, State-Action-Reward-State-Action (SARSA), Actor-Critic, and Deep Q-Network (DQN). Besides the three common ML algorithms mentioned above, there are also some special algorithms like semi-supervised learning, transfer learning, ensemble learning, and multi-task learning that are often combined with basic algorithms to achieve more efficient and accurate prediction or decision-making in practical applications [221].

### Application of ML methods based on omics technologies in discrimination and diagnosis of LTBI

LTBI is a condition in which individuals are infected with MTB but do not exhibit any clinical symptoms or imaging characteristics. The discrimination and diagnosis of LTBI cannot rely on medical image data and clinical information data because there are no clinical signs or symptoms. Therefore, the application of ML techniques in the discrimination and diagnosis of LTBI can only be achieved by mining biomarker data, which primarily consists of various omics data such as transcriptomics, proteomics, and metabolomics. Herein, we focus on elucidating the application of ML methods based on omics technologies in the discrimination and diagnosis of LTBI.

#### Transcriptomics

Host cells infected with MTB induce host-specific immune responses, resulting in differential mRNA and non-coding RNA expressions in immune cells and other cells at different stages of MTB infection. Transcriptomics provides a major tool for understanding how these cellular processes happen [235]. Transcriptomics analysis primarily consists of two most useful methods for whole transcriptome gene expression profiling: microarray and RNA sequencing (RNA-seq) [236, 237]. Microarray is based on the hybridization of predetermined labeled probes with target cDNA sequences, while RNA-seq directly sequences cDNA chains using sequencing technology. With the continuous development of ML technology, artificial intelligence analysis based on microarray and RNA-seq data has emerged, providing a new perspective for the discrimination and diagnosis of LTBI (Table 3).

**Table 3:** List of studies on ML methods based on transcriptomics technology in the differential diagnosis of LTBI and ATB

Study	Sample size	Countries	Methods^ψ^	Biomarkers	ML methods	Sensitivity	Specificity	Accuracy	AUC
Lee et al. [238]	TB (*n* = 15), LTBI (*n* = 17), HCs (*n* = 15)	Taiwan, China	Microarray	PTPRC + ASUN + DHX29 + NEMF	Decision tree, random forest, support vector machine, Bayesian best	97.9% with PTPRC + ASUN + DHX29 under the Bayesian model	Unknown	97.8%	0.979
Lu et al. [239]	Discovery cohort, TB (*n* = 4), LTBI (*n* = 4), HCs (*n* = 4); qPCR validation cohort, TB (*n* = 25), LTBI (*n* = 36), HCs (*n* = 22); additional validation cohort, TB (*n* = 17), LTBI (*n* = 19)	China	Microarray	CXCL10, ATP10A, and TLR6 combination	Decision trees and unsupervised cluster analysis	71% in the additional validation cohort	89% in additional validation cohort	Unknown	Unknown
Wang et al. [240]	Identification cohort, ATB (*n* = 28), LTBI (*n* = 25), HCs (*n* = 31); validation cohort, ATB (*n* = 51), LTBI (*n* = 44), HCs (*n* = 35)	China	RNA-seq	TNFRSF10C, IFNG, PGM5, EBF3, A2ML1	Decision trees and unsupervised cluster analysis	86.2% with a combination of TNFRSF10C, EBF3, and A2ML1	94.9%	87.8%	Unknown
Maertzdorf et al. [241]	ATB patients (*n* = 120), LTBI (*n* = 60), HCs (*n* = 20); external cohorts, from the Gambia (*n* = 75), from the Uganda (*n* = 62)	Africa	RT-PCR	GBP1, IFITM3, P2RY14, and ID3	Random forest, decision tree	Using a cutoff of 0.8, Uganda: 73%, Gambia: 85%; using a cutoff of 0.6, Uganda: 87%, Gambia: 88%	Using a cutoff of 0.8, Uganda: 78%, Gambia: 76%; using a cutoff of 0.6, Uganda: 75%, Gambia: 68%	82% in Uganda and 89% in Gambia	AUC = 0.89 in Gambia and AUC = 0.82 in Uganda
Bayaa et al. [242]	ATB patients (*n* = 141), LTBI (*n* = 26), HCs (*n* = 71)	Multiple countries	qRT-PCR	RISK6	No	90.9%	88.5%	Unknown	0.930
Gong et al. [243]	ATB patients (*n* = 51), lung cancer (*n* = 30), INFLA (*n* = 30), HCs (*n* = 15)	China	qRT-PCR	SERPING1, BATF2, UBE2L6, and VAMP5	No	88%	78%	Unknown	0.840

^ψ^Methods used for screening and identification of biomarkers

*A2ML1* alpha-2-macroglobulin like protein 1, *ASUN* asunder spermatogenesis regulator, *ATB* active tuberculosis, *ATP10A* ATPase phospholipid transporting 10A, *AUC* area under curve, *BATF2* basic leucine zipper transcription factor, *CXCL10* chemokine (C-X-C motif) ligand 10, *DHX29 DEAH* (Asp-Glu-Ala-His) box polypeptide 29, *EBF3* early B cell factor 3, *GBP1* guanylate binding protein 1, *HCs* health controls, *ID3* inhibitor of DNA binding 3, *IFITM3* interferon-induced transmembrane protein 3, *IFNG* interferon-γ gene, *INFLA* patients with pneumonia, *LTBI* latent tuberculosis infection, *ML* machine learning, *NEMF* nuclear export mediator factor, *P2RY14* UDP-glucose-specific G(i) protein-coupled P2Y receptor, *RT-PCR* reverse transcription-polymerase chain reaction, *PGM5* phosphoglucomutase 5, *PTPRC* protein tyrosine phosphatase receptor type C, *qRT-PCR* quantitative real-time PCR, *RNA-seq* RNA-sequencing, *RISK6* six whole blood gene transcriptomic signature, *SERPING1* serpin peptidase inhibitor C1 inhibitor member 1, *TNFRSF10C* TNF receptor superfamily member 10C, *TLR* Toll-like receptor, *TB* tuberculosis, *UBE2L6* ubiquitin-conjugating enzyme E2L6, *VAMP5* vesicle-associated membrane protein 5

##### Microarray

Microarray-based ML methods offer a promising approach to accurately differentiate between ATB and LTBI. In a prospective cohort study, differential gene expression analysis using microarray and quantitative reverse transcription polymerase chain reaction was conducted to identify biomarkers relevant to the differentiation of ATB and LTBI [238]. The analysis identified 4 biomarkers: nuclear export mediator factor, asunder spermatogenesis regulator, DEAH (Asp-Glu-Ala-His) box polypeptide 29, and protein tyrosine phosphatase receptor type C. To verify their potential, a naive Bayes model was employed using asunder spermatogenesis regulator, DEAH (Asp-Glu-Ala-His) box polypeptide 29, and protein tyrosine phosphatase receptor type C, producing a highly sensitive (97.9%) and accurate (97.8%) diagnostic model [238]. However, it should be noted that the performance of this model has not been validated in external cohorts.

To address the limitation of lacking external validation, Lu et al. [239] conducted a study in which they performed microarray analysis on gene expression profiles from 3 groups: TB, LTBI, and healthy controls (HCs). Through this analysis, they discovered 3 genes [chemokine (C-X-C motif) ligand 10 (CXCL10), ATPase phospholipid transporting 10 A (ATP10A), and TLR6] and developed an LTBI diagnostic model using a decision tree algorithm [239]. The model they constructed exhibited a sensitivity of 71% and specificity of 89% in the external validation cohort, consisting of 42, 55, and 22 individuals from the respective groups [239]. It is important to note that the initial discovery cohort had a relatively small sample size of only 4 individuals per group, which may have impacted the precision of the model [244]. Having a sufficient sample size is crucial for the reliability, generalizability, and statistical power of a diagnostic model. Firstly, a larger sample size allows the model to encounter a greater variety of examples and learn more representative patterns from the data, reducing the risk of overfitting and coincidental performance [245]. Secondly, it strengthens the statistical power, enabling more accurate estimation of the model’s performance metrics and enhancing the ability to identify subtle patterns or differences [246]. This also leads to smaller confidence intervals for performance metrics, enabling more precise estimation of the model’s true performance [247]. Lastly, having an adequate sample size within different populations or specific patient subgroups ensures robust and reliable estimation of the model’s performance within each subgroup, which is crucial for understanding the model’s effectiveness across the entire target population. Therefore, researchers constructing an LTBI diagnostic model should carefully consider the sample size requirements based on the problem’s complexity, population diversity, and statistical considerations to ensure the accuracy and reliability of the model.

##### RNA-seq

RNA-seq technology offers several advantages over microarray analysis, including high throughput, sensitivity, and the ability to detect new genes and genetic variations [248]. Utilizing RNA-seq analysis, researchers have successfully identified biomarkers to differentiate between ATB and LTBI, employing advanced ML algorithms to analyze and classify differentially expressed genes. In the study conducted by Wang et al. [240], RNA-seq technology was used in conjunction with unsupervised classification of genes obtained through PPD stimulation in 3 groups: ATB, LTBI, and HCs. The study identified a 3-gene signature set comprising TNF receptor superfamily member 10C (TNFRSF10C), early B cell factor 3 (EBF3), and alpha-2-macroglobulin like protein 1 (A2ML1), which achieved a correct classification rate of 91.5% in distinguishing between the different groups, with a high sensitivity of 86.2% and specificity of 94.9%. To validate the diagnostic performance of the 3-gene signature set, an application cohort of 147 subjects with suspected ATB was utilized. In this cohort, the 3-gene set demonstrated a sensitivity of 82.4% and specificity of 92.4% for detecting ATB [240]. It is noteworthy that the combination of multiple biomarkers and ML algorithms has significantly improved the diagnostic performance of LTBI. However, caution must be exercised in interpreting these research results, as all studies mentioned were conducted in the same region (China), and the LTBI diagnostic model constructed may not necessarily apply to populations with different genetic backgrounds. Furthermore, the relatively small sample sizes used in the 2 microarray-based studies warrant validation of the diagnostic models in large-scale cohort studies to assess generalizability [238, 239].

The use of ML algorithms in identifying biomarkers that can distinguish between ATB and LTBI is promising for clinical practice. However, the sensitivity and specificity of biomarkers may vary in different populations, and more research is required to confirm their clinical utility [249]. Nonetheless, the potential of RNA-seq technology combined with advanced ML algorithms in identifying relevant biomarkers for distinguishing between ATB and LTBI offers promise for the accurate diagnosis and treatment of LTBI.

##### Real-time polymerase chain reaction (RT-PCR)

The development of LTBI diagnostic models using microarray and RNA-seq technologies can be challenging due to their complexity and high cost, limiting their implementation in economically underdeveloped regions. However, recent research has shown that RT-PCR, coupled with ML algorithms, can offer an affordable and user-friendly alternative to enhance LTBI diagnosis. In a study conducted in Bangalore, India, RT-PCR and ML algorithms were utilized to model gene expression data [241]. This approach resulted in a 4-gene combination consisting of guanylate binding protein 1 (GBP1), interferon-induced transmembrane protein 3 (IFITM3), UDP-glucose-specific G(i) protein-coupled P2Y receptor (P2RY14), and inhibitor of DNA binding 3 (ID3), which achieved promising diagnostic performance. In the Gambian validation group, the 4-gene combination had an area under the curve (AUC) of 0.89, a sensitivity of 85%, and a specificity of 76%. In the Ugandan cohort, it achieved an AUC of 0.82, a sensitivity of 73%, and a specificity of 78% [241]. This study is notable for its relatively large sample sizes of ATB, LTBI, and HCs populations within the cohort, ensuring the accuracy of identifying and validating potential biomarkers. Moreover, the research demonstrated the favorable sensitivity, specificity, and diagnostic accuracy of the LTBI diagnostic model across different national cohorts, highlighting its potential applicability in diverse countries.

Indeed, not all transcriptomic studies require the integration of ML algorithms to achieve good diagnostic capability for LTBI. Some studies have demonstrated favorable results by solely utilizing transcriptomic analyses to discover potential biomarkers and their application in LTBI discriminatory diagnosis. In one prospective case–control study, a whole blood gene transcriptomic signature called six whole blood gene transcriptomic signature, consisting of guanylate binding protein 2, Fc fragment of IgG receptor 1b, serpin peptidase inhibitor C1 inhibitor member 1, tubulin gamma complex associated protein 6, tRNA methyltransferase 2 homolog A, and short chain dehydrogenase/reductase family 39U member 1, was identified using transcriptomics [242]. The six whole blood gene transcriptomic signature combination achieved an impressive AUC of 0.930, with a sensitivity of 90.9% and specificity of 88.5% in distinguishing between ATB and LTBI [242]. In another study, researchers integrated differentially expressed genes, co-expression networks, and short-term sequence analyses [243]. They mined transcriptome data in the Gene Expression Omnibus database and identified 4 biomarkers (UBE2L6, BATF2, SERPING1, and VAMP5). The combination of these biomarkers achieved a diagnostic sensitivity of 88% and specificity of 78% for ATB, which was significantly higher than the sensitivity of 75.3% and specificity of 69.1% achieved using T-SPOT detection [243]. These examples highlight the potential of transcriptomic analyses alone in identifying and utilizing biomarkers for LTBI discriminatory diagnosis, yielding encouraging diagnostic performance.

##### Single-cell RNA sequencing (scRNA-seq)

scRNA-seq is a powerful tool that allows for in-depth exploration of cellular heterogeneity and gene expression profiles at a single-cell resolution [250–253]. While scRNA-seq is primarily used to study gene expression patterns and cellular dynamics in various diseases, it also holds the potential to aid in the diagnosis and understanding of TB [254, 255]. By utilizing scRNA-seq, researchers can assess the transcriptomic features of individual immune cells within LTBI patients, providing detailed information on cell composition, functional status, and immune responses related to LTBI [256]. The following are potential applications of scRNA-seq in the diagnosis of LTBI. (1) Identification of LTBI-specific gene expression features [257, 258]. scRNA-seq can help identify gene expression patterns and molecular characteristics specific to LTBI. By comparing the transcriptomic profiles of immune cells between LTBI patients and individuals without LTBI or with ATB, researchers may discover differentially expressed genes or gene modules indicative of LTBI. (2) Characterization of immune cell subpopulations [259–261]. scRNA-seq facilitates the identification and characterization of various immune cell subpopulations associated with LTBI. Through analysis of individual cell transcriptomes, researchers can delineate distinct immune cell clusters that emerge during LTBI, including T cells, B cells, macrophages, DCs, and more. This enables a deeper understanding of the cellular dynamics, interactions, and functional states of these immune cells during LTBI. (3) Assessment of immune cell activation and response [261, 262]. scRNA-seq can unveil the activation status and functional responses of immune cells during LTBI. By studying gene expression profiles, researchers can identify specific cell subpopulations and pathways involved in the host immune response to MTB infection. This helps elucidate immune mechanisms associated with LTBI and has the potential to identify targets for diagnostics or therapeutic interventions. It is worth noting that the application of scRNA-seq in the diagnosis of LTBI is still in its early stages, and further research is required to fully explore its potential. ML models based on scRNA-seq technology for LTBI diagnosis also require further investigation.

#### Proteomics

Proteomics and ML algorithms have shown great potential in identifying various biomarkers for distinguishing between LTBI and ATB. Studies have focused on using MTB-specific proteins, host-specific antibodies, and cytokines as potential biomarkers (Table 4).

**Table 4:** List of studies on ML methods based on proteomics technology in the differential diagnosis of LTBI and ATB

Study	Simple size	Countries	Biomarkers	ML methods	Sensitivity	Specificity	AUC
Li et al. [263]	Discovery cohort: ATB (*n* = 52), LTBI (*n* = 37), HCs (*n* = 27); validation cohort: ATB (*n* = 205), LTBI (*n* = 123), HCs (*n* = 112);	China	Rv0934, Rv1827, Rv1860, and Rv3881c	Cluster analysis	67.3%	91.2%	Unknown
Li et al. [264]	Discovery cohort: ATB (*n* = 60), LTBI (*n* = 60), HCs (*n* = 60); validation cohort: ATB (*n* = 100), LTBI (*n* = 100), HCs (*n* = 100)	China	Rv1860, RV3881c, Rv2031c, and Rv3803c	Random forest	93.3% in training cohort and 95% in validation cohort	97.7% in training cohort and 80% in validation cohort	0.981 in training cohort and 0.949 in validation cohort
Cao et al. [265]	Training cohort, ATB (*n* = 20), LTBI (*n* = 20); validation cohort, ATB (*n* = 92), LTBI (*n* = 93), HCs (*n* = 94)	China	Rv1408, R0248, Rv2026c, Rv2716, Rv2031c, Rv2928, and Rv2121c	Logistic regression and hierarchical clustering	96.77% in training cohort and 93.33% in validation cohort	93.75% in training cohort and 93.1% in validation cohort	0.9844 in training cohort and 0.9810 in validation cohort
Peng et al. [266]	TBI (*n* = 100), LTBI (*n* = 60), HCs (*n* = 44)	China	15 MTB antigen-specific antibodies	Logistic regression model and hierarchical clustering	85.4%	90.3%	0.944
Delemarre et al. [267]	Discovery cohort: ATB (*n* = 20), LTBI (*n* = 40), HCs (*n* = 20); validation cohort: ATB (*n* = 12 + 31), LTBI (*n* = 20 + 20)	USA	CCL1, CXCL10, VEGF, and ADA2	Logistic regression	95% in discovery cohort, 75% and 100% in validation cohort 1 and 2	90% in discovery cohort, 100% and 30% in validation cohort 1 and 2	Unknown
Luo et al. [268]	Training cohort: ATB (*n* = 468), LTBI (*n* = 424); Test set, ATB (*n* = 121), LTBI (*n* = 102); validation cohort: ATB (*n* = 125), LTBI (*n* = 138)	China	ESAT-6, CFP-10, IFN-γ, ESR, Hs-CRP	Random forest and bagged ensemble algorithms	98.85% in Training cohort; 93.39% in Test set; 92.80% in validation cohort	95.65% in training cohort; 91.18% in Test set; 89.86% in validation cohort	0.995 in training cohort; 0.978 in Test set; 0.963 in validation cohort
Morris et al. [269]	Discovery cohort: TB (*n* = 146), LTBI (*n* = 146) other diseases (OD) (*n* = 146); validation cohort: TB (*n* = 122), OD (*n* = 127)	Sub-Saharan Africa	Fibrinogen, alpha-2-macroglobulin, CRP, MMP-9, transthyretin, complement factor H, IFN-γ, IP-10, and TNF-α	Random forest and logistic regression	92% in the test set	71% in the test set	0.84
Agranoff et al. [270]	Training cohort: ATB (*n* = 102), HCs (*n* = 91); validation cohort: ATB (*n* = 77), HCs (*n* = 79)	UK	Transthyretin, C-reactive protein, Neopterin, and serum amyloid A	Support vector machine and tree classification	93.5%	94.9%	Unknown
Luo et al. [271]	Discovery cohort: ATB (*n* = 50), LTBI (*n* = 49), HC (*n* = 50); validation cohort: ATB (*n* = 28), LTBI (*n* = 24), HCs (*n* = 26)	China	Eotaxin, MDC, and MCP-1	No	87.76%	91.84%	0.94

*ATB* active tuberculosis, *LTBI* latent tuberculosis TB infection, *ML* machine learning, *AUC* area under curve, *HCs* healthy controls, *MTB Mycobacterium tuberculosis*, *CCL* chemokine (C–C motif) ligand, *CXCL10* chemokine (C-X-C motif) ligand 10, *VEGF* vascular endothelial growth factor, *ADA2* adenosine deaminase 2, *ESAF-6* early secretary antigenic target-6, *CFP-10* culture filtrate protein-10, *IFN-γ* interferon-γ, *ESR* erythrocyte sedimentation rate, *Hs-CRP* high-sensitivity C-reactive protein, *MMP-9* matrix metalloprotein-9, *IP-10* interferon-γ inducible protein-10, *TNF-α* tumor necrosis factor-α, *MDC* myeloid dendritic cell, *MCP-1* human macrophage chemoattractant protein-1

##### MTB-specific proteins

Selecting specific antigen targets from the 4000 encoded proteins of MTB to accurately distinguish LTBI is a challenging task [272]. As mentioned before, the TST uses MTB PPD, which cannot distinguish interference from BCG vaccination and environmental mycobacterial infections, thus reducing its diagnostic specificity. Although the IGRA has improved by utilizing RD1 antigens ESAT-6 (Rv3875) and CFP-10 (Rv3874), effectively eliminating interference from BCG and most environmental mycobacterial infections, they still cannot differentiate LTBI from ATB. Therefore, the development of new LTBI discrimination models must focus on selecting antigens associated with RD-LTBI [13]. Some studies have utilized ML algorithms to classify and combine MTB-specific proteins, thereby improving diagnostic performance and identifying optimal protein combinations [263–265]. In a cohort study involving 440 samples, microarray analysis, clustering, and protein–protein interaction network identified 4 biomarkers: Rv0934, Rv1827, Rv1860, and Rv3881c [263]. These biomarkers showed 67.3% sensitivity and 91.2% specificity in distinguishing ATB from LTBI. ELISA validation demonstrated a sensitivity of 71.22% and specificity of 91.87% for diagnosing ATB [263]. Another study replaced Rv0934 and Rv1827 with Rv2031c and Rv3803c and analyzed the combinations of Rv1860, Rv3881c, Rv2031c, and Rv3803c using a random forest model [264]. This approach improved the sensitivity (93.3%) and specificity (97.7%) for discriminating ATB from LTBI in the training and validation sets [264]. Both these studies used discovery and validation cohorts from the same nationality (China) and had similar sample sizes. However, there were significant differences in the sensitivity and specificity of the constructed LTBI diagnostic models, which may be attributed to differences in MTB antigen selection and ML algorithm choice. Regarding antigen selection, Li et al. [263] chose 4 MTB antigens, namely Rv0934, Rv1827, Rv1860, and Rv3881c, while Li et al. [264] selected the following 4 MTB antigens: Rv1860, RV3881c, Rv2031c, and Rv3803c. Therefore, assuming that the ML algorithm has no impact on these diagnostic models, incorporating Rv2031c and Rv3803c may enhance the diagnostic performance of the models. However, it is worth noting that different ML algorithms can significantly affect the diagnostic performance of models, further complicating the issue.

Unlike the above-mentioned studies using 4 MTB antigens and a single ML algorithm, Cao et al. [265] expanded the number of MTB antigens to 7 (Rv1408, R0248, Rv2026c, Rv2716, Rv2031c, Rv2928, Rv2121c) when constructing their discriminative LTBI diagnostic model. Additionally, they used both logistic regression and hierarchical clustering as ML algorithms. The combination of these 7 antigens achieved the best differentiation between ATB and LTBI in the training set with an AUC of 0.9844, sensitivity of 96.77%, and specificity of 93.75%. This combination had an AUC of 0.9632, sensitivity of 93.33%, and specificity of 93.1% in the validation set [265].

These studies indicate that proteomic approaches based on MTB antigens provide new avenues and methods for early LTBI diagnosis. ML algorithms can further enhance the accuracy of these methods. However, the available candidate antigens for LTBI discrimination are currently limited, and many immunological characteristics and diagnostic performance of RD-LTBI-related antigens remain unknown. This limits the diversity of LTBI diagnostic models constructed using proteomic approaches based on MTB antigens. Moreover, increasing the number of MTB antigens does not necessarily lead to a significant improvement in the performance of the diagnostic model, as seen in the aforementioned studies. Therefore, when constructing LTBI diagnostic models, the selection of MTB antigens needs to consider a comprehensive balance between diagnostic ability and health economics factors. We should ensure optimal diagnostic performance while minimizing the number of antigens included in the model. This can enhance the practical feasibility and cost-effectiveness of the model.

##### Host-specific antibodies, and cytokines

In addition to MTB antigen biomarkers, host-specific antibodies and cytokines have emerged as potential biomarkers for TB diagnosis. Several studies have explored the use of host biomarkers, such as antibodies and cytokines, to discriminate between ATB and LTBI [266, 268, 271]. One study utilized a serum proteomics microarray analysis and identified a combination of 15 TB antigen-specific antibodies that demonstrated high sensitivity (85.4%) and specificity (90.3%) in distinguishing between ATB and LTBI [266]. However, this study lacked validation using other technologies, and the complexity of the 15-antibody combination may limit its cost-effectiveness and practicality. Another approach involved employing logistic regression algorithms to obtain a cytokine combination of chemokine (C–C motif) ligand 1, CCL10, vascular endothelial growth factor, adenosine deaminase 2 [267]. This combination achieved high sensitivity (95%) and specificity (90%) in the discovery cohort for discriminating between ATB and LTBI [267]. However, the validation cohorts showed varying specificities, likely due to differences in infection states, countries, races, and ages within the studied population [267]. While this model shows promise, extensive experimental validation is needed before its widespread implementation.

Moreover, the use of multiple proteins and cytokines as biomarkers to differentiate between different MTB infection states holds great potential. Multiple studies have identified various cytokines as discriminative biomarkers and have employed ML algorithms, such as random forests, support vector machines, tree classification, single-layer perceptrons, and multilayer perceptrons, to validate them, achieving high sensitivity and specificity. For instance, a study conducted in sub-Saharan Africa identified 9 proteins as potential discriminative biomarkers, achieving a sensitivity of 92% and a specificity of 71% [269]. Another prospective study utilized proteomic fingerprinting and ML algorithms to determine a four-protein combination that yielded a diagnostic accuracy of 94% using support vector machines, regardless of HIV status [270]. In China, a combination of multiple biomarkers (ESAT-6, CFP-10, IFN-γ, ESR, Hs-CRP) was used to establish a diagnostic model employing random forests and bagging algorithms [268]. This model demonstrated sensitivity and specificity values of 92.80% and 89.86%, respectively, for discriminating between ATB and LTBI [268]. Similarly, another study utilized the T-SPOT.TB test to identify a three-factor combination with an AUC of 0.94 and a sensitivity and specificity of 87.76% and 91.84%, respectively [271].

These studies highlight the potential for utilizing multiple cytokine protein combinations and ML algorithms in identifying LTBI. Such an approach holds promise in terms of operational efficiency and cost-effectiveness as a discriminatory method. However, further research, data analysis, and empirical validation are necessary to optimize their value before they can be effectively implemented.

#### Critical factors in ML-based differential diagnosis of ATB and LTBI

In the previous section, we discussed various methods for data collection used in building ML algorithms, including microarray, RNA-seq, scRNA-seq, and proteomics. We also evaluated the sensitivity, specificity, and accuracy of the diagnostic model. However, there are several other factors to consider when developing ML models for discriminating between ATB and LTBI. (1) Algorithm selection. The choice of ML algorithm is crucial for the accuracy and efficiency of the diagnostic model. Support vector machine, decision trees, random forest, and logistic regression are commonly used algorithms for LTBI discrimination models. It is important to carefully assess each algorithm’s advantages, limitations (Table 5), and its ability to handle high-dimensional data, interpretability, and generalizability. (2) Sample size. Adequate sample size is essential for the development and validation of ML models. Studies with small sample sizes may encounter issues such as overfitting or limited generalizability. Acquiring LTBI samples can be challenging due to the difficulty in identifying LTBI individuals without characteristic clinical and radiological features. Additionally, the high cost of omics technologies makes large-scale sequencing difficult for many researchers. (3) Feature selection. ML models require relevant features that effectively differentiate between ATB and LTBI. In addition to gene expression profiles and protein markers, clinical features like age, gender, history of TB exposure, chest X-rays, CT scans, and TST or IGRA results should also be considered. However, including irrelevant or redundant features may degrade model performance. The optimal features should be selected based on appropriate ML algorithms. (4) Model validation. Model validation is essential for building a robust model. Cross-validation, bootstrapping, or an independent test set can be used to evaluate the model’s performance, generalizability, and robustness. The model’s stability against changes in training and test datasets should be assessed. (5) Subject inclusion and exclusion criteria. In constructing LTBI discrimination models, the inclusion and exclusion criteria for subjects like ATB, LTBI, and HCs may vary across different countries. This can result in heterogeneity of results, even with the same sample size, algorithms, and evaluation metrics, thereby affecting the model’s performance. Considering these additional factors will provide researchers with a comprehensive understanding of the advantages and limitations of ML-related studies in discriminating between ATB and LTBI, leading to a stronger evaluation of the current status in this field.

**Table 5:** Advantages and disadvantages of the most common algorithms used in LTBI differential diagnostic models

Algorithm	Advantages	Disadvantages
Support vector machine (SVM)	Good generalization ability for high dimensional and nonlinear problems; Can adapt to different data types by selecting different kernel functions; Performs well with a small amount of data	Long training time for large-scale datasets; Challenging to select the appropriate kernel function and parameters for noisy data and nonlinear problems; Does not provide direct probability estimates
Decision trees	Easy to understand and interpret; Can handle nonlinear features and large-scale data; Suitable for both classification and regression problems; Minimal data preprocessing is required	Prone to overfitting, especially with deep trees; Performs poorly with continuous and highly correlated features
Random forest	High accuracy; Can handle high-dimensional and large-scale datasets; Robust to noise and missing data; Provides feature importance estimation	A more complex model with longer training time; Substantial memory consumption for datasets with large feature spaces; Less effective for highly correlated features
Logistic regression	Simple and fast computation; Interpretable parameter weights to understand feature importance; Suitable for binary classification problems	Performs poorly with nonlinear relationships in the data; Prone to underfitting; May not perform well with high-dimensional data or highly correlated features
Hierarchical clustering	No need to specify the number of clusters in advance; Provides a hierarchical structure of clusters; Works with numerical and categorical data; Allows for visual analysis through dendrograms	High computational complexity; Difficulty with high-dimensional data; Restrictions on data types; Irreversible clustering results

## The implementation significance, advantages, and disadvantages of ML in the differential diagnosis of LTBI

The implementation significance of ML in the identification and diagnosis of LTBI lies on its ability to extract features from a large amount of data through algorithmic training, effectively recognizing the degree of interaction between different variables, and forming a structured knowledge system to provide personalized decision support and rapid diagnosis [11, 273]. This approach can shorten diagnosis time, improve accuracy, and better support healthcare resource allocation and decision-making. Compared to traditional methods, ML in LTBI identification and diagnosis has significant advantages. (1) Collection and processing of large amounts of data for learning. ML is capable of collecting and processing large amounts of data and continuously improving model accuracy and precision [274, 275]. (2) Independent decision-making from supervisory personnel. ML models can make decisions independently, without relying on subjective judgment and experience from supervisory personnel [276]. (3) Strong adaptability. ML models continually learn and adapt to new data, which provides more accurate predictions and diagnoses. (4) More comprehensive and systematic data management and analysis. ML is based on big data analysis, which allows for more comprehensive analysis and management of data to discover functional interactions between data, improving diagnostic accuracy [277]. (5) Ability to handle complex data relationships. ML can learn and predict different types of multidimensional and complex data relationships [278].

However, the application of ML in LTBI diagnosis also faces some challenges. (1) It requires a large amount of data support. The application of ML technology requires a large amount of data support to model training, and the lack of complete, accurate and effective data hinders the application of the model [279]. (2) Poor interpretability. While achieving the best prediction and diagnosis results, ML lacks intuitive interpretability, making it difficult for people to fully understand and accept the results [280]. (3) Dependence on algorithms and technologies. The algorithms and technologies of ML are constantly evolving, and their results are subject to the current technology and algorithm selection, which affects the accuracy of obtaining false-normal or disease information. (4) Data privacy and protection issues. The application of ML requires a large amount of data circulation, which poses data leakage and personal data privacy protection issues. (5) Uncertainty in feature extraction and selection. ML models are often based on feature extraction and selection to describe patient data, and how to effectively select features becomes an important problem in modeling that requires further research.

In summary, ML technology presents promising implementation significance in identifying and diagnosing LTBI. However, several technical issues and limitations need to be addressed to meet clinical needs. When implementing ML technology, it’s essential to consider its advantages and disadvantages comprehensively, leading to the constant improvement of techniques and models to achieve better application results based on 4 criteria: correctness, robustness, readability, and temporality [230].

## Future directions of ML for the differential diagnosis of LTBI

With the advancement of modern medical technology, ML is becoming an important tool in the field of identifying and diagnosing LTBI. It can quickly identify the interactions between different variables, provide personalized and timely decision support, and discover hidden patterns and associations in the case of data through the analysis of large amounts of data [234, 281]. As technology continues to evolve, future research will focus on integrating different types of data sources, improving model interpretability, developing intelligent evaluation models, establishing large-scale data repositories, and developing standardized clinical diagnostic and treatment protocols. These developments will further enhance the application and effectiveness of ML in the identification and diagnosis of LTBI, thus providing better medical services to patients.

### Combining different types of data sources

Combining different types of data sources is an important direction for the development of ML in the differential diagnosis of LTBI. Currently, the main data sources used by ML include medical imaging data, biomarker data, and clinical information data. Medical imaging data including chest X-rays, CT scans, MRI, and other medical images, is one of the main data sources for ML. ML technology can extract features required to accurately diagnose ATB from these imaging data, such as pulmonary texture, pulmonary structure, and lesion morphology [220, 222]. In contrast to ATB, differential diagnosis of LTBI cannot be completed based solely on imaging data as individuals with LTBI do not exhibit significant differences compared to healthy individuals. Therefore, medical imaging data must be combined with other data sources for differential diagnosis. Biomarker data is the most important source for using ML methods to diagnose LTBI [282]. Previous research has found significant differences between individuals with LTBI and individuals with ATB or healthy individuals in various biomarkers [13, 67, 283, 284]. Biomarker data in latent TB diagnosis is obtained through biological experiments and includes information on molecules, cells, tissues, and fluids like blood, urine, sputum, and saliva. ML can be utilized to discover features unique to the group with LTBI, build models, and carry out differential diagnoses. Clinical information data is another important source of data, which includes basic patient information, medical history, clinical manifestations, laboratory test results, and other relevant information [285, 286]. While these data sources alone are not sufficient for the precise identification of LTBI, they provide critical information for diagnosis and treatment. If combined with biomarker data and transformed into mathematical models using ML methods, they can be used for the differential diagnosis of LTBI.

Future research should focus on how to combine medical imaging data, biomarker data, and clinical information data to improve the efficacy of ML in the differential diagnosis of LTBI. Firstly, the joint model approach can be used to combine different types of data in the same model for learning, which can optimize the predictive results of different data types. For example, federated learning of clinical information data and biomarker data can better identify the biochemical characteristics of the TB population, thereby improving diagnostic accuracy [287]. Secondly, the cascade learning approach categorizes different types of data into different sub-tasks, which are then cascaded for learning and prediction in a specific sequence, thus improving the overall predictive accuracy [288, 289]. For example, medical imaging data and clinical information data can be analyzed and predicted first, followed by the use of biomarker data to further refine and diagnose the LTBI, improving its differential diagnostic performance. Finally, ensemble learning methods can be used to combine multiple different types of learning algorithms at the resulting level [290]. For instance, medical imaging data and biomarker data can be used to train different models separately, and then the prediction results of different models are fused using ensemble learning methods to obtain the final differential diagnosis result of LTBI. In conclusion, combining different types of data sources is a crucial direction for the development of ML in the differential diagnosis of LTBI. By utilizing various techniques, ML can integrate medical imaging data, biomarker data, and clinical information data to improve the accuracy and efficacy of differential diagnosis of LTBI.

### Improve the interpretability of the LTBI diagnostic model

Improving the interpretability of the model is another important direction for the development of ML in the differential diagnosis of LTBI [291, 292]. Traditionally, ML algorithms often use black-box models to balance interpretability and predictiveness [293]. However, for clinical medicine, interpretability models are preferred as they can provide more information to doctors, help them make the right decisions, and also comply with the reality that clinical decisions are determined by multiple factors. In interpretable models, each element of the model results should have a clear meaning so that doctors can clearly understand which features have the greatest impact on the results. Currently, the main methods to improve the interpretability of ML models are as follows. (1) Visualization. This method uses visualization techniques to transform complex models and data into intuitive and easy-to-understand data visualization results. For example, using t-distributed Stochastic Neighbor Embedding or PCA to reduce dimensionality and explore relationships between variables. (2) Feature importance assessment. This method can find the features with the most informative value for the model’s prediction results and derive feature importance scores. For example, using decision trees, random forests, and other algorithms to compute feature importance scores can help doctors understand how the model results are affected by different features. (3) Local interpretability methods. This method can provide interpretable information for individual data analysis needs. For example, using local interpretable model-agnostic explanations to explain complex models as more easily understandable local models. (4) Symbolic analysis and rule extraction. This method produces interpretable predictive models such as rules, decision tables, and decision trees. For example, using classification-based rule algorithms, such as iterative dichotomiser 3, C4.5 (an extension of ID3 algorithm), and classification and regression tree, to generate interpretable rules. In summary, improving the interpretability of the model is an important direction for the development of ML in the differential diagnosis of LTBI. By using different methods, ML can improve the interpretability of the model, allowing doctors to better understand the predictive results of ML algorithms and further help them make more accurate diagnostic decisions.

### Development of an intelligent model for differential diagnosis of LTBI

Future ML methods need to be improved in evaluating the differential diagnosis of LTBI in order to achieve automation and semi-automation of evaluations [294]. On the one hand, automated evaluations can generate suggestions and diagnostic reports for differential diagnosis of LTBI through guidance and analysis of ML models and provide timely treatment plans and patient management. On the other hand, semi-automated evaluation platforms can utilize various medical knowledge bases and rules to determine the output results of ML models, providing more credible and objective decision results, enabling doctors to provide recommended solutions, and supporting the updating of patient treatment plans.

### Establishment of a large-scale TB professional data warehouse

A large-scale TB-specific data warehouse can offer a deeper understanding of the differential diagnosis and treatment options for LTBI. By mining tens of thousands of case data, it is possible to better understand the natural correlations between different malignant events. Therefore, the establishment of large-scale data warehouses will play an increasingly important role in the future [295, 296]. The steps to achieve big data processing include: 1) integrating data sources, collecting large amounts of data into a structured data warehouse, including medical history, clinical information, diagnostic results, and treatment methods; 2) data cleaning and quality control, such as missing value filling, outlier handling, and data format conversion; 3) developing critical feature extraction and data mining algorithms, extracting the necessary information from the data to assist doctors in diagnosis and treatment; and 4) analyzing and visualizing big data, discovering hidden patterns, correlations, and predictive results, which can provide more early diagnosis information for doctors. In the future, these data warehouses will become more detailed and comprehensive, and the application of big data will continuously improve the accuracy and efficiency of ML algorithms, providing better decision support for clinical doctors.

### Developing standardized clinical diagnostic and treatment regimens for LTBI

To better apply ML technology and incorporate it into clinical practice, it is necessary to develop standardized clinical diagnostic and treatment regimens for LTBI [297]. This includes creating effective and standardized practice guidelines for discriminating diagnosis of LTBI to ensure the stability and predictive ability of the model. Authentication and recognition of research findings are prerequisites and foundations for developing these standardized guidelines. Future development requires broad and deep communication and cooperation among doctors, medical technicians, researchers, and decision-makers in policy to ensure that every stage is fully considered and discussed in detail. By developing standardized diagnostic and treatment regimens, ML technology will better serve clinical practice and achieve higher quality, reliability, and precision in discriminating diagnosis of LTBI.

## Conclusions

Despite the availability of various diagnostic tools for ATB, there is still no reliable method for differential diagnosis of LTBI. TB is a highly prevalent global disease, and early discrimination of LTBI is crucial in its control and treatment. However, discriminative diagnosis of LTBI remains a challenge as none of the currently available diagnostic tools can differentiate between LTBI and ATB. Although 3 new TST methods (C-TB, Diaskintest, and EC skin test) and 7 latest IGRAs (AdvanSure™ TB-IGRA ELISA, Wantai TB-IGRA, Standard E TB-Feron, QIAreach QFT, ichroma™ IGRA-TB, VIDAS™ TB-IGRA, and T-Track^®^ TB) have shown excellent performance in diagnosing ATB, they are unable to discriminate ATB from LTBI.

To identify potential biomarkers for the differential diagnosis of LTBI, we conducted an in-depth analysis of the immunological mechanisms responsible for LTBI mediated by MTB. Although MTB infection remains poorly understood, we explore essential aspects of innate and adaptive immunity, signaling pathways, MTB immune escape mechanisms, the MTB energy source, and its regulatory mechanisms, as well as epigenetic and gene regulation. By understanding these factors’ roles and interactions, we hope to uncover potential biomarkers or diagnostic tools that can more accurately and efficiently detect LTBI.

In recent years, the emergence and rapid development of ML algorithms have provided new approaches for the discrimination diagnosis of LTBI and ATB. Medical image data, biomarker data, and clinical information data are the main data sources used by ML. However, since the LTBI population does not exhibit any clinical symptoms or imaging manifestations, the discrimination diagnosis of LTBI is only feasible through mining biomarker data. Biomarker data mainly comprise MTB and host biomarkers, and the ML algorithms used for the discrimination diagnosis of LTBI and ATB are based on their transcriptomics and proteomics data. Multiple studies have shown that the use of ML algorithms significantly improves the sensitivity, specificity, and diagnostic efficiency of discriminating between LTBI and ATB, and a combination of multiple biomarkers can further enhance performance.

Despite the advantages of ML, such as improved sensitivity, specificity, and diagnostic efficiency, it poses some limitations that need to be addressed. These limitations include the need for a large amount of data support, poor interpretability, dependence on algorithms and technologies, issues related to data privacy and protection, and uncertainty in feature extraction and selection. In the future, research should consider combining different types of data sources, improving model interpretability, developing intelligent discrimination diagnosis models, establishing large-scale TB specialty data warehouses, and developing standardized clinical diagnostic and treatment regimens for LTBI. The application of ML in the early diagnosis and prevention of TB and LTBI represents a promising approach to accurately discriminate and diagnose LTBI and ATB. Furthermore, it can reduce the progression of LTBI to ATB and contribute to the WHO’s goal of eliminating TB by 2035.

## Data Availability

Not applicable.

## Abbreviations

AC: Autoencoder
ATB: Active tuberculosis
AUC: Area under the curve
BCG: Bacillus Calmette–Guérin
CA: Cluster analysis
CFP-10: Culture filtrate protein-10
CTL: Cytotoxic T lymphocyte
DCs: Dendritic cells
ELISA: Enzyme-linked immunosorbent assay
ESAT-6: Early secreted antigen target-6
GM-CSF: Granulocyte–macrophage colony-stimulating factor
HCs: Health controls
HIV: Human immunodeficiency virus
IFN-γ: Interferon-γ
IGRAs: Interferon-gamma release assays
IL: Interleukin
LPS: Lipopolysaccharides
LTBI: Latent tuberculosis infection
MHC II: Histocompatibility complex II
ML: Machine learning
MS: Malate synthases
MTB: *Mycobacterium tuberculosis*
NK cells: Natural killer cells
PAMPs: Pathogen-associated molecular patterns
PPD: Purified protein derivative
PRRs: Pattern recognition receptors
RF: Random forest
RISK-6: Six gene expression features
ROS: Reactive oxygen species
TB: Tuberculosis
TBF: TB-Feron
TNF-α: Tumor necrosis factor-α
TST: Tuberculin skin test
TRIF: Toll/interleukin 1 receptor-domain-containing adapter-inducing interferon-β
WHO: World Health Organization
